# Primary cross-resistance to BRAFV600E-, MEK1/2- and PI3K/mTOR-specific inhibitors in BRAF-mutant melanoma cells counteracted by dual pathway blockade

**DOI:** 10.18632/oncotarget.6600

**Published:** 2015-12-14

**Authors:** Ilaria Penna, Alessandra Molla, Giulia Grazia, Loredana Cleris, Gabriella Nicolini, Federica Perrone, Benedetta Picciani, Michele Del Vecchio, Filippo de Braud, Roberta Mortarini, Andrea Anichini

**Affiliations:** ^1^ Human Tumors Immunobiology Unit, Fondazione IRCCS Istituto Nazionale dei Tumori, Milan, Italy; ^2^ Department of Experimental Oncology and Molecular Medicine, Fondazione IRCCS Istituto Nazionale dei Tumori, Milan, Italy; ^3^ Laboratory of Molecular Pathology, Department of Pathology and Laboratory Medicine, Fondazione IRCCS Istituto Nazionale dei Tumori, Milan, Italy; ^4^ Medical Oncology Unit 1, Department of Medical Oncology, Fondazione IRCCS Istituto Nazionale dei Tumori, Milan, Italy

**Keywords:** melanoma, BRAF, MEK, PI3K/mTOR, apoptosis

## Abstract

Intrinsic cross-resistance to inhibition of different signaling pathways may hamper development of combinatorial treatments in melanoma, but the relative frequency of this phenotype and the strategies to overcome this hurdle remain poorly understood. Among 49 BRAF-mutant melanoma cell lines from patients not previously treated with target therapy, 21 (42.9%) showed strong primary resistance (IC_50_ > 1 μM) to a BRAFV600E inhibitor. Most of the BRAF-inhibitor-resistant cell lines showed also strong or intermediate cross-resistance to MEK1/2- and to PI3K/mTOR-specific inhibitors. Primary cross-resistance was confirmed in an independent set of 23 BRAF-mutant short-term melanoma cell cultures. MEK1/2 and PI3K/mTOR co-targeting was the most effective approach, compared to BRAF and PI3K/mTOR dual blockade, to counteract primary resistance to BRAF inhibition and the cross-resistant phenotype. This was shown by extensive drug interaction analysis, tumor growth inhibition assays *in-vivo*, p-ERK and p-AKT inhibition, promotion of melanoma apoptosis, apoptosis-related protein modulation, activation of effector caspases and selective modulation of genes involved in melanoma drug resistance and belonging to the ERK/MAPK and PI3K/AKT canonical pathways. Compared to co-targeting of mutant BRAF and PI3K/mTOR, the association of a MEK1/2 and a PI3K/mTOR inhibitor was more effective in the activation of Bax and of caspase-3 and in the induction of caspase-dependent melanoma apoptosis. Furthermore Bax silencing reduced the latter effects. These results suggest that intrinsic resistance to BRAF inhibition is frequently associated with primary cross-resistance to MEK and PI3K/mTOR blockade in BRAF-mutant melanoma and provide pre-clinical evidence for a combinatorial approach to counteract this phenotype.

## INTRODUCTION

Inhibitors of BRAFV600E have changed the clinical management of patients with BRAF-mutant advanced melanoma, since significant improvements in progression-free survival (PFS) and in overall survival (OS) have been reported in Phase 3 trials [1–2]. These inhibitors can induce objective responses or stabilization of disease in a high fraction of patients [2–3], although relapse occurs due to adaptive [4] or acquired [5] resistance mechanisms. In addition, ∼20% of patients show primary/intrinsic resistance to BRAF inhibitors and experience tumor progression at first assessment during therapy [2–3]. Lack of response has been observed even in trials with MEK inhibitors as Trametinib and Selumetinib/AZD6244 [6–7], pointing to primary resistance also to the targeting of MEK. To counteract resistance to BRAF inhibitors, several clinical trials based on combinatorial targeting of BRAF and MEK have been carried out recently, and results have indicated a significant improvement in PFS and (in one study) in OS, compared to monotherapy with the BRAF inhibitor alone [8–10]. However, strikingly, in all these studies ∼30% of the patients showed progressive disease at 6 months of treatment [8–10], pointing to the potential role of cross-resistance mechanisms. Indeed, primary cross-resistance to BRAF and MEK inhibitors has been documented in a subset of melanomas, where it is related to the MITF profile [11–13], and in cell lines [14].

In the attempt to address all these limitations associated with targeting of a single (MAPK) pathway, several pre-clinical studies have suggested that combinatorial targeting of MAPK and PI3K/mTOR intracellular pathways may be a potential approach to improve target therapy of melanoma and to overcome resistance and cross-resistance mechanisms [14–17]. However, key questions to be answered are whether BRAF-mutated melanomas, with intrinsic resistance to BRAF and/or MEK inhibitors, also have primary cross-resistance to PI3K/mTOR inhibitors and which is the frequency of such cross-resistant phenotype. A second issue to be addressed is whether combinatorial targeting of different signaling pathways is effective even in the tumors with primary cross-resistance.

Recently, we observed instances of primary cross-resistance to a MEK1/2 (AZD6244) and to a dual PI3K/mTOR (BEZ235) inhibitor in some melanoma cell lines (18). Starting from this initial evidence, in this study we assessed responsiveness to BRAFV600E-, MEK1/2-, dual PI3K/mTOR- and dual mTORC1/2-specific inhibitors in 49 BRAF-mutant melanoma cell lines and in an independent set of 23 BRAF-mutant short-term melanoma cell cultures, all isolated from patients never treated with target therapy. Among cell lines with strong intrinsic resistance to the BRAF inhibitor (IC_50_ > 1 μM, *n* = 21), 81% (seventeen) showed strong or intermediate cross-resistance to the MEK1/2- and the PI3K/mTOR-specific inhibitors. Extensive drug interaction analysis on all 49 cell lines and mechanistic studies in cross-resistant cell lines indicated that co-targeting of MEK1/2 and PI3K/mTOR, *in-vitro* and *in-vivo*, was a more effective combinatorial treatment, compared to co-targeting of BRAF and PI3K/mTOR, to counteract the primary cross-resistant phenotype.

## RESULTS

### Primary resistance to BRAFV600E inhibition is associated with cross-resistance to MEK1/2 and PI3K/mTOR inhibitors in BRAF-mutant melanoma cells

**Figure 1:**
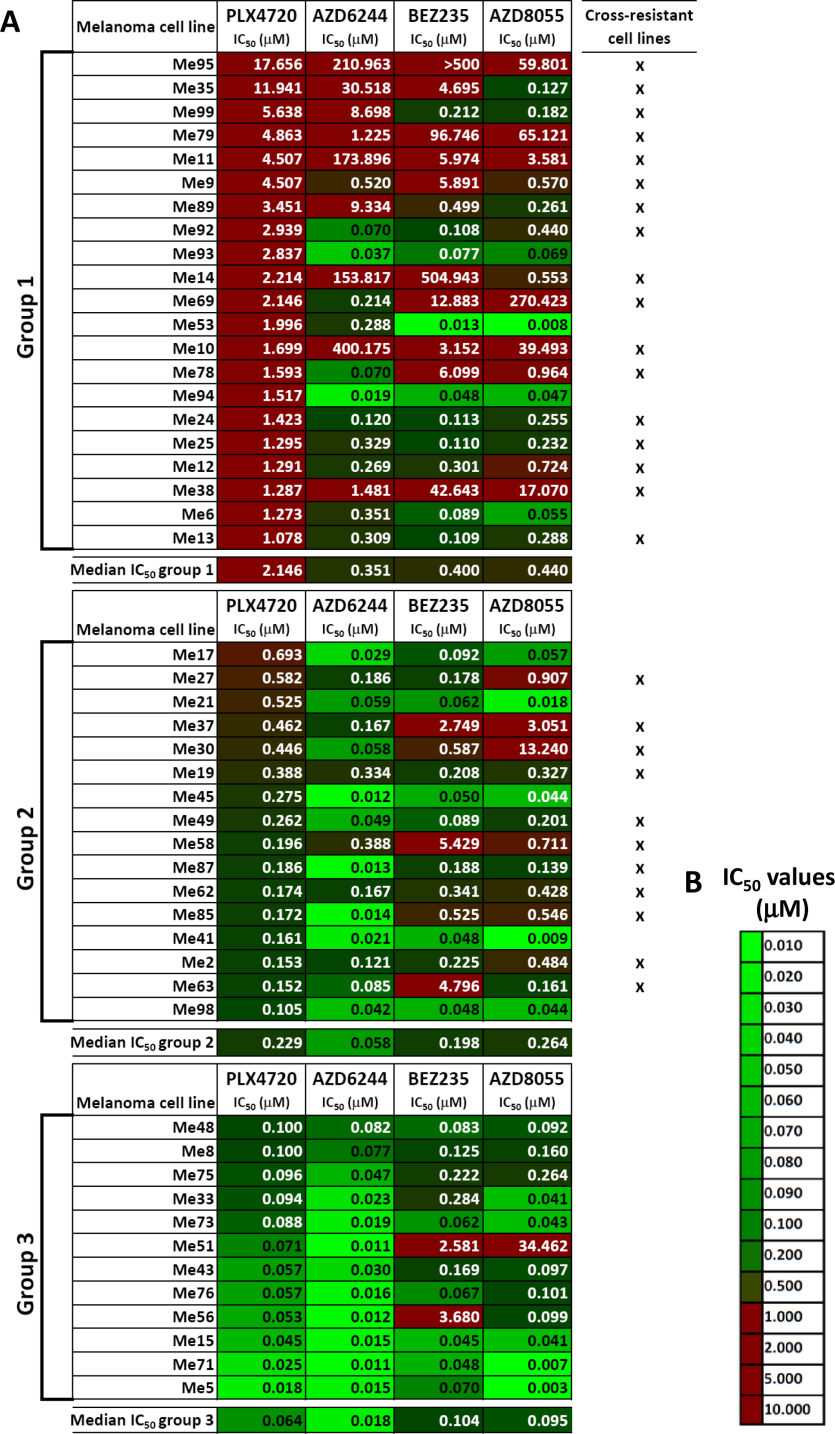
Responsiveness to BRAF-V600E-, MEK1/2- or PI3K/mTOR-specific inhibitors in BRAF-mutant melanoma cell lines (**A**) Susceptibility to PLX4720 (BRAFV600E inhibitor), AZD6244 (MEK1/2 inhibitor), BEZ235 (dual PI3K/mTOR inhibitor) and AZD8055 (dual mTORC1/2 inhibitor), shown as IC_50_ values (μM), was assessed by a 72 h MTT assay in a panel of 49 melanoma cell lines. IC_50_ values obtained through non linear regression analysis of twelve-point dose-response curves spanning 4 logs of inhibitor concentrations. Melanomas were distinguished into three groups after ranking based on PLX4720 IC_50_ values (group 1: IC_50_ > 1 μM; group 2: IC_50_ > 0.1 μM but < 1 μM; group 3: IC_50_ ≤ 0.1 μM). (**B**) Color code used for highlighting differences in IC_50_ values in panel A.

**Figure 2:**
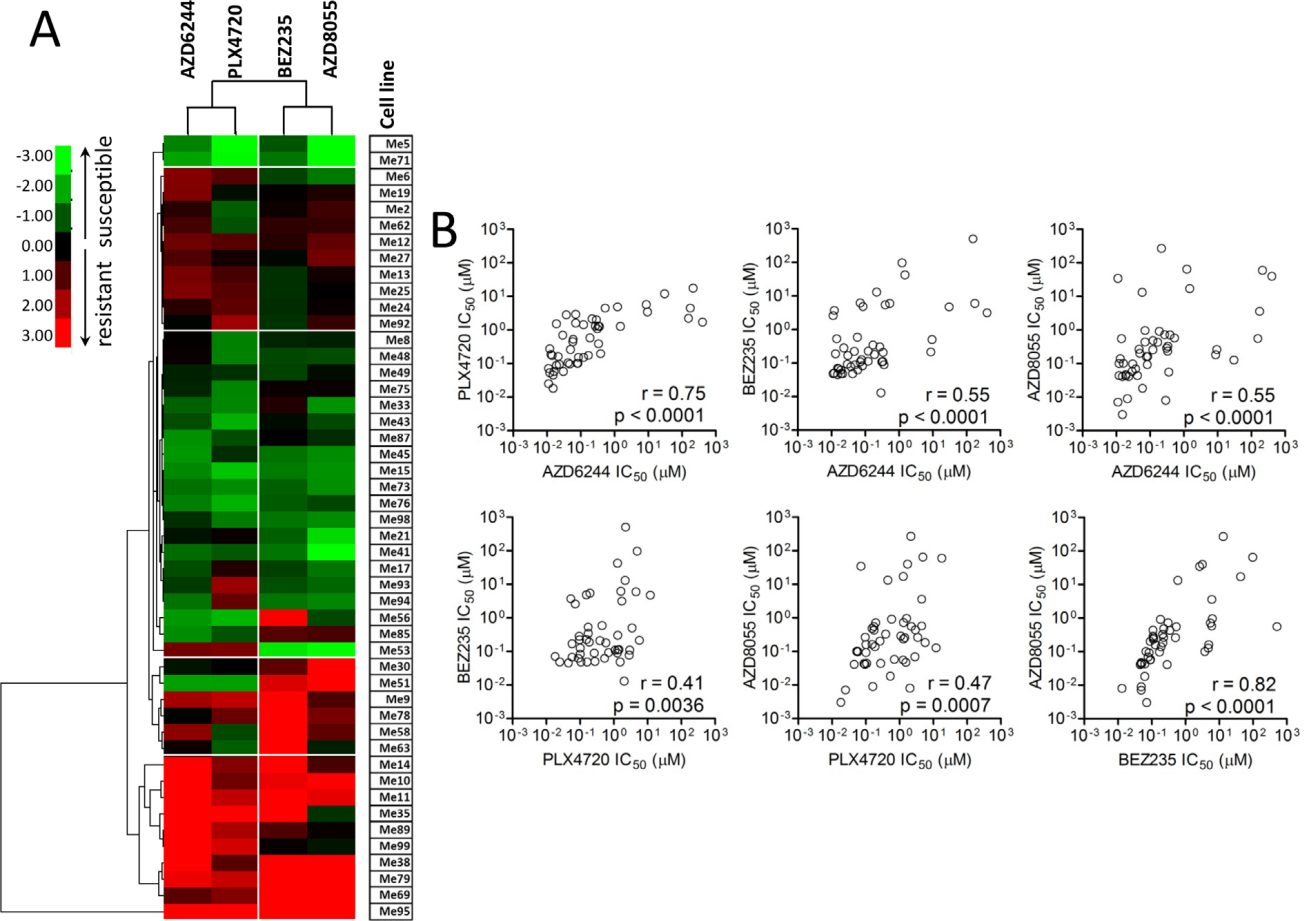
Responsiveness profiles of BRAF-mutant human melanoma cell lines to BRAFV600E-, MEK1/2-, and PI3K/mTOR-specific inhibitors are significantly correlated (**A**) Hierarchical clustering of normalized and Log-transformed IC_50_ values for the indicated inhibitors in 49 melanoma cell lines. Each IC_50_ value (as listed in Figure 1) was normalized against the median IC_50_ value of each inhibitor in the whole panel. (**B**) Spearman correlation analysis of IC_50_ values for each of the six possible combinations of the four inhibitors in the whole panel of melanoma cell lines.

We used 49 BRAF-mutant melanoma cell lines isolated from surgical specimens of patients not previously treated with BRAFV600E inhibitors, nor with any other target-specific inhibitor, to test responsiveness to BRAFV600E (PLX4720), MEK1/2 (AZD6244), dual PI3K/mTOR (BEZ235) and dual mTORC1/2 (AZD8055) inhibitors (Figure 1A). Three susceptibility groups were defined by ranking cell lines based on their IC_50_ values for PLX4720. Strong resistance (IC_50_ > 1 μM) was found in 21/49 cell lines (42.9%, group 1), intermediate resistance (IC_50_ = 0.1 to 1 μM) and susceptibility (IC_50_ < 0.1 μM) to PLX4720 were observed in 16/49 (32.7%, group 2) and 12/49 cell lines (24.5%, group 3), respectively (Figure 1A and Supplementary Table 1D for descriptive statistics). Seventeen out of 21 melanoma lines (marked with an “x” in Figure 1A) classified in group 1, showed either strong or intermediate primary resistance to MEK1/2 and PI3K/mTOR inhibitors. Strong or intermediate cross-resistance to PI3K/mTOR inhibitors was also found in 11/16 cell lines in group 2, while only two cell lines in group 3 (PLX4720 susceptible) showed strong resistance to the PI3K/mTOR inhibitors (Figure 1A). Hierarchical clustering of log-transformed and normalized IC_50_ values confirmed the existence of distinct subsets of cell lines with cross-resistance to all inhibitors or susceptible to all of them (Figure 2A). Spearman correlation analysis of IC_50_ values for all six possible combinations of four inhibitors showed that all the susceptibility profiles were significantly correlated (Figure 2B).

**Figure 3:**
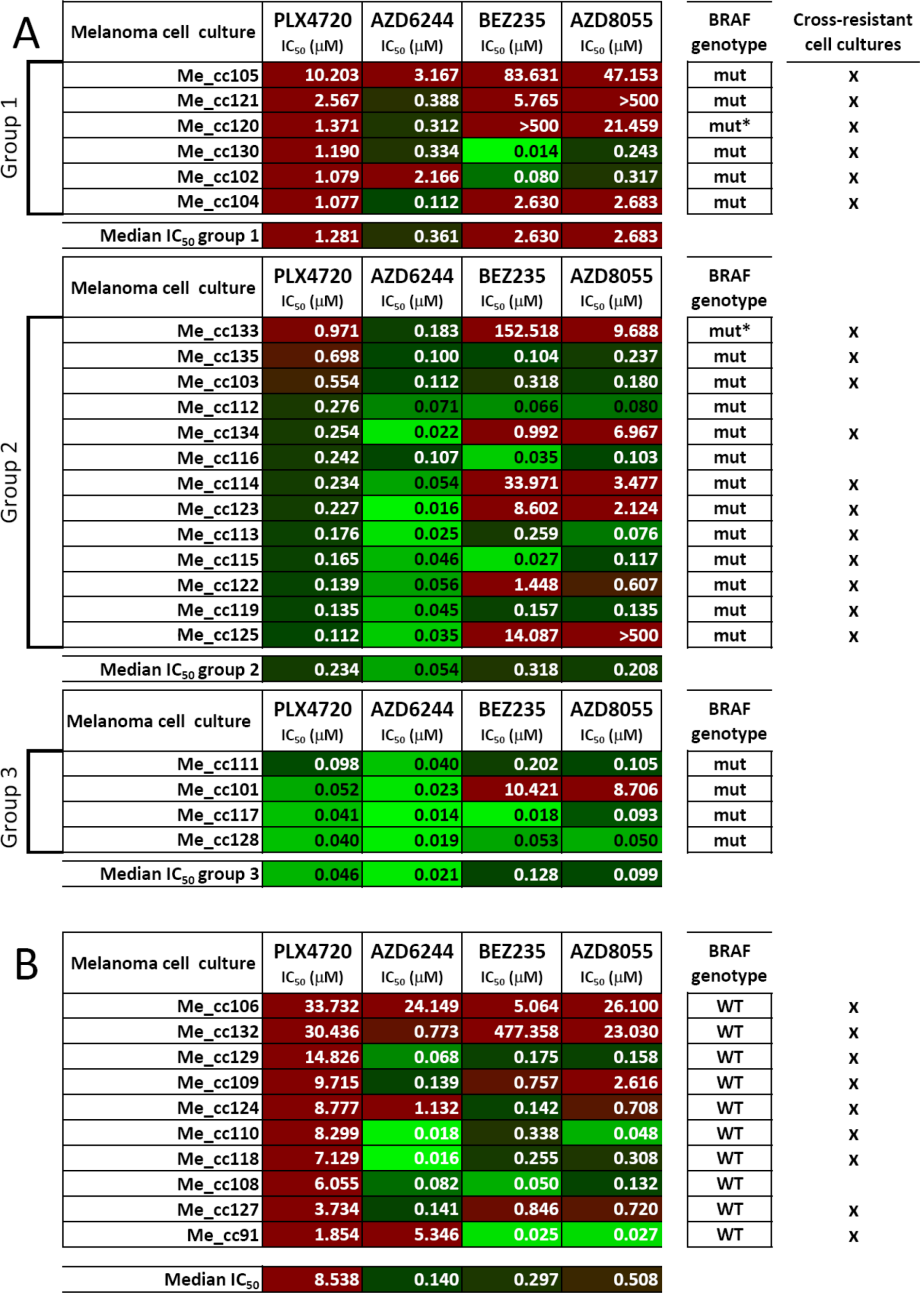
Responsiveness to BRAF-V600E-, MEK1/2- or PI3K/mTOR-specific inhibitors in short-term melanoma cell cultures (**A**, **B**) Susceptibility to PLX4720, AZD6244, BEZ235 and AZD8055, shown as IC_50_ values (μM), was assessed as described in the legend to Figure 1, in a panel of 33 melanoma cell cultures (Me_cc) bearing mutant BRAF (*n* = 23, A) or wt BRAF (*n* = 10, B). Melanoma cell cultures were established from surgical specimens of lymph node metastases and were tested between the third and fifth *in-vitro* passage. Short term melanoma cell cultures from BRAF-mutant lesions were distinguished into three groups after ranking based on PLX4720 IC_50_ values as in Figure 1. BRAF genotype: mut: BRAFV600E; mut*: BRAFV600K WT. BRAF wild type. IC_50_ values were highlighted by the color code indicated in Figure 1B.

To validate these findings, an independent panel of short-term melanoma cell cultures (all tested between the third and fifth *in-vitro* passage), obtained from 23 BRAF-mutant metastatic specimens of patients not previously treated with target-specific inhibitors, was used to test responsiveness to the same set of inhibitors. The same classification into three subsets based on ranking of PLX4720 IC_50_ values was applied. We found that 6/6 PLX4720-resistant melanoma cell cultures (group 1) showed strong (i.e. IC_50_ > 1 μM) or intermediate (i.e. IC_50_ > 0.1 μM) cross-resistance to MEK1/2 and PI3K/mTOR inhibitors, and 11/13 cultures in group 2 (intermediate resistance to PLX4720) showed also strong or intermediate cross-resistance to PI3K/mTOR inhibitors (Figure 3A). As a control, 10 short-term melanoma cell cultures from tumors with wt BRAF were characterized for responsiveness to the four inhibitors. As expected [19], all the BRAF wt melanoma cell cultures were strongly resistant to PLX4720, but some of them also showed strong resistance to the MEK1/2 or to the PI3K/mTOR inhibitors (Figure 3B). Interestingly, the melanoma cell culture Me_cc135, with intermediate cross-resistance, was isolated from a specimen of a patient who subsequently (4.4 months after Me_cc135 isolation) was treated with a BRAF inhibitor and underwent progressive disease after two cycles of therapy. In contrast, melanoma cell cultures Me_cc111 and Me_cc128, with a cross-susceptible phenotype, were isolated from patients who subsequently (75.4 and 2.8 months, after Me_cc111 and Me_cc128 isolation, respectively) were treated with the association of a BRAF and a MEK inhibitor or in monotherapy with a MEK inhibitor and experienced a partial response or a complete response, respectively.

Twelve days clonogenic assays on representative cell lines (Me43 and Me71) and short-term melanoma cell cultures (Me_cc117 and Me_cc128) from the cross-susceptible group 3 (Supplementary Figure 1A), indicated a strong suppression of melanoma growth by AZD6244, PLX4720, BEZ235 and AZD8055, often detected at the lowest inhibitor dose (0.1 μM). In contrast, clonogenic assays on representative cell lines (Me35, Me6, Me13) and short-term melanoma cell cultures (Me_cc102) from group 1 (Supplementary Figure 1B) showed a partial or markedly reduced inhibitory effect by AZD6244 (on Me35 and Me_cc102), by PLX4720 (on Me35, Me6, Me13 and Me_cc102), and by AZD8055 (on Me35, Me13 and Me_cc102). BEZ235 exerted a reduced inhibitory effect on Me35, even at the highest dose, in agreement with the high IC_50_ value in this cell line (Supplementary Figure 1B). Taken together, these assays confirmed that cell lines and short-term melanoma cell cultures in group 1 showed markedly reduced responsiveness to multiple inhibitors.

The panel of 49 melanoma cell lines shown in Figure 1, was further characterized for several molecular or phenotypic features associated with drug resistance [20–23], but no significant association was found, between the drug susceptibility groups and: a) the PTEN, MDM4 and MDM2 expression levels; b) the constitutive p-ERK, p-AKT and p-S6 levels (Supplementary Table 1A–1C and 1E–1G). We also assessed the MITF phenotype of the cell lines and short-term melanoma cell cultures, as either high or low expression of this transcription factor has been associated with drug resistance in melanoma [11–13]. We found that melanoma cell lines retained the MITF phenotype of the corresponding lesions, but both MITF^hi^ and MITF^lo^ cell lines and short term cultures were found in each of the three susceptibility groups (data not shown).

Taken together, these results indicated that intrinsic resistance to BRAFV600E inhibition can be frequently associated with cross-resistance to MEK1/2 and/or PI3K/mTOR inhibitors in BRAF-mutant melanoma cells.

### Co-targeting of MAPK and PI3K/mTOR pathways in melanoma cells with a primary cross-resistant phenotype has synergistic effects *in vitro* and anti-tumor activity *in vivo*

**Figure 4:**
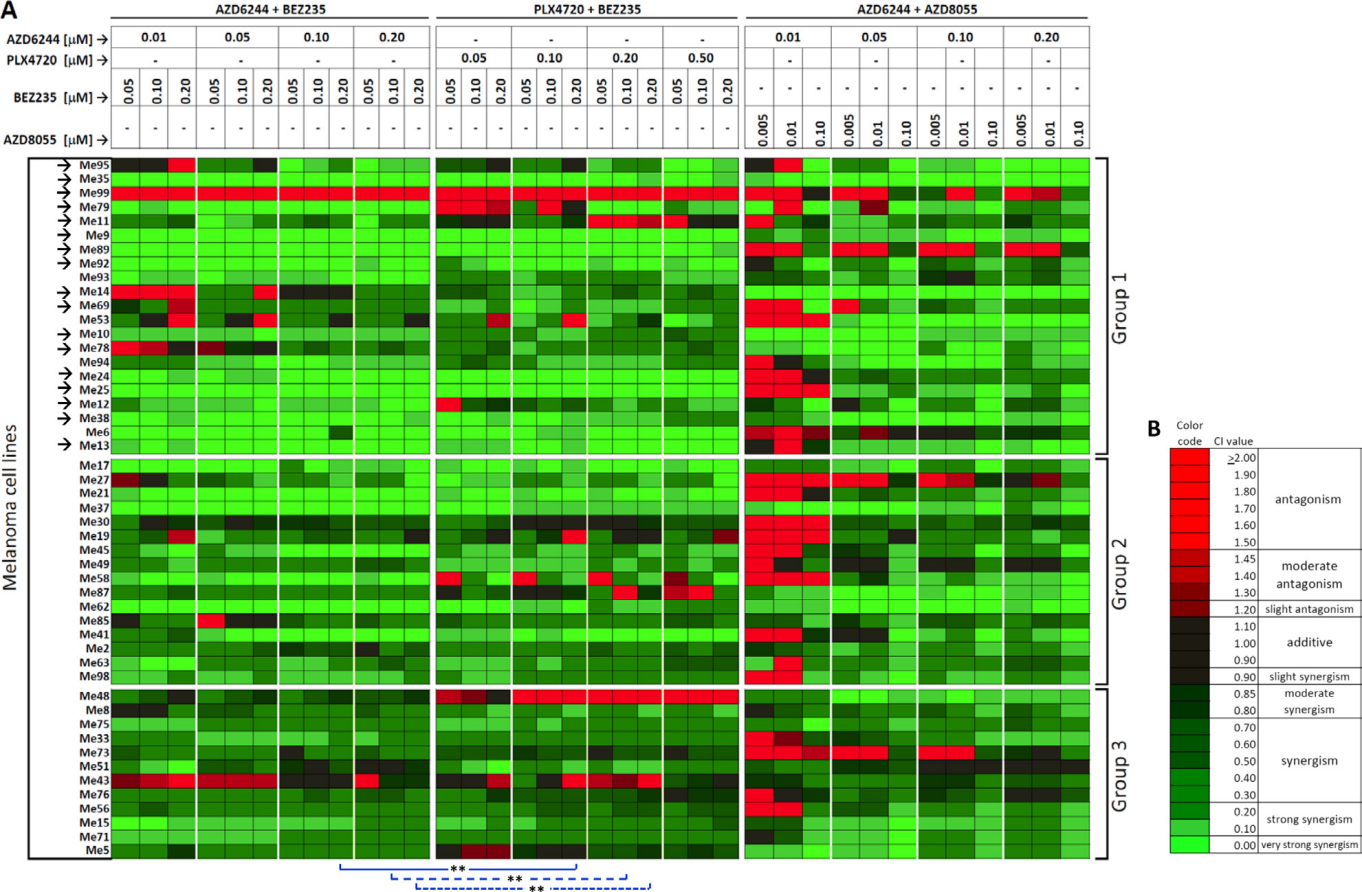
Synergistic drug interaction by co-targeting of MAPK and PI3K/mTOR pathways in melanoma cell lines (**A**) Drug interaction analysis by the association of AZD6244 and BEZ235, PLX4720 and BEZ235, or AZD6244 and AZD8055 was carried out by the Chou-Talalay method in three groups of melanoma cell lines with different responsiveness to PLX4720 (Group 1, Group 2 and Group 3 as defined in Figure 1). Results for each of the indicated combinations of inhibitors (drug doses listed at the top of the Figure), for each cell line, are shown as Combination Indexes (CI) values by a color code shown in panel (B) Arrows: cell lines (*n* = 17) with a strong cross-resistant phenotype as in Figure 1. Blue lines: Wilcoxon matched pair test of CI values observed by AZD6244-BEZ235 vs. PLX4720-BEZ235 combinations having equivalent drug dosing. **p < 0.01. (**B**) color code for CI values, range of values and corresponding meaning (antagonism, additivity, synergy) according to ref. 24. Red indicates antagonism, green indicates synergism.

To test whether combinatorial targeting of MAPK and PI3K/mTOR pathways could overcome primary cross-resistance, we performed drug interaction analysis by the Chou-Talalay method [24]. To this end, 49 cell lines belonging to the three susceptibility groups were treated with AZD6244-BEZ235, or PLX4720-BEZ235 or AZD6244-AZD8055 associations. For each association of inhibitors, twelve different combinations of doses (indicated at the top of Figure 4A) were evaluated, yielding a 1,764-point drug interaction matrix (49 cell lines by 36 drug combinations). Strong synergistic effects, documented by CI values < 0.3 (Figure 4A and 4B for color codes and meaning of CI values and Supplementary Table 2 for primary data), were achieved on almost all cell lines from group 2 and 3. Interestingly, strong synergism was observed at most drug dosing combinations, against 20/21 melanoma cell lines in group 1 (Figure 4A), including 16/17 lines with the cross-resistant phenotype (Figure 4A, *arrows*). By clonogenic assays (Supplementary Figure 2A, 2B) AZD6244-BEZ235 and PLX4720-BEZ235 combinatorial treatments exerted a strong inhibitory effect on melanoma growth not only on two cell lines from group 3 (Me43 and Me71), but also on three cell lines from group 1 (Me35, Me6, Me13).

In addition, the AZD6244-BEZ235 combination induced a significantly stronger synergistic effect (lower CI values) against the whole panel of 49 melanoma cell lines compared to PLX4720-BEZ235, in three out of nine experimental conditions where both AZD6244 and PLX4720 were present at equivalent doses (Figure 4A, statistical comparisons highlighted by blue lines and asterisks).

In addition to a combination index matrix, the drug interaction analysis yielded also a 1,764-point Fraction Affected (FA) matrix (Supplementary Figure 3A, 3B for color coding of FA values and Supplementary Table 3 for primary data). Analysis of the FA matrix led to two main conclusions. First, combinatorial treatment with AZD6244-BEZ235 or PLX4720-BEZ235, but to a much lesser extent with AZD6244-AZD8055, allowed to achieve FA values > 0.50, or even > 0.70, on most cross-resistant cell lines in group 1. Second, and most relevant, in each of the 9 matched drug combinations (identified by matched symbols at bottom of Supplementary Figure 3A) where AZD6244 and PLX4720 were used at equivalent doses, AZD6244-BE235 induced significantly higher FA values compared to PLX4720-BEZ235 on the 21 cell lines in group 1, characterized by strong primary resistance to PLX4720 and by frequent cross-resistance (Supplementary Figure 4A, 4B for statistical analysis).

We then compared anti-tumor activity *in-vivo* of AZD6244-BEZ235 and PLX4720-BEZ235 combinatorial treatments. To this end, we established a SCID mouse model based on s.c. xenograft of a cell line from group 1 (Me13). This cell line was characterized by strong primary resistance to PLX4720 (IC_50_ > 1 μM) and by intermediate resistance to AZD6244 and BEZ235 (IC_50_ > 0.1 μM). Both treatments exerted a significant inhibitory effect on tumor growth, compared to control animals treated with vehicle, but the AZD6244-BEZ235 association was significantly more effective than PLX4720-BEZ235 treatment (Supplementary Figure 5). Moreover, no evident signs of treatment-related toxicity were observed (data not shown).

Taken together, the *in-vitro* and *in-vivo* experiments indicated that co-targeting of MEK1/2 and PI3K/mTOR has improved anti-tumor activity compared to co-targeting of mutant BRAF and PI3K/mTOR even in melanoma cells with an intrinsic cross-resistant phenotype.

### MEK1/2 and PI3K/mTOR co-targeting is more effective than BRAF and PI3K/mTOR dual blockade in inhibition of signaling molecule phosphorylation in PLX4720-resistant and in cross-resistant melanoma cell lines

**Figure 5:**
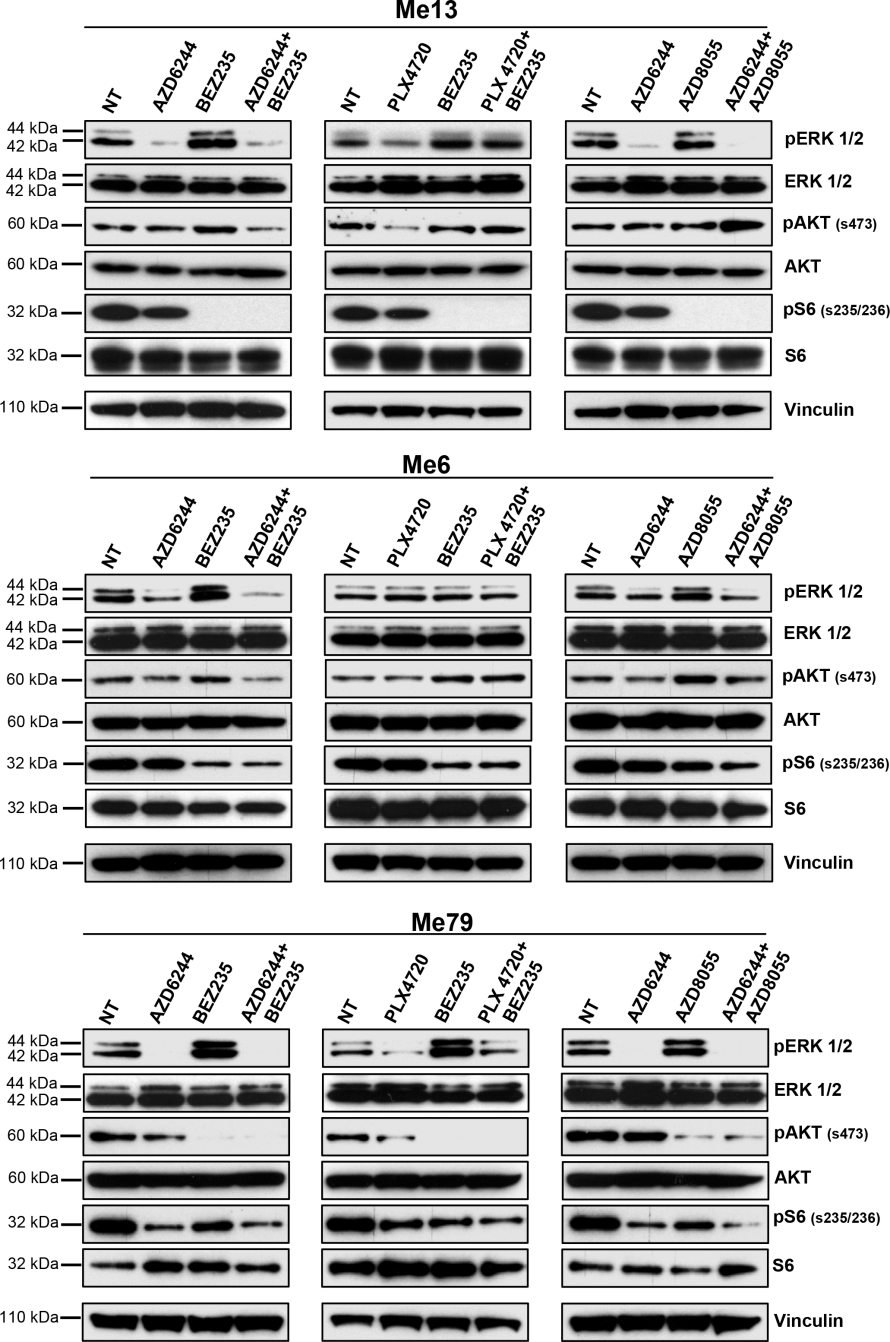
Inhibition of p-ERK and p-AKT by combinatorial treatments in melanoma cells with intrinsic resistance to PLX4720 Three melanoma cell lines from group 1 (Me13, Me6 and Me79) were treated O/N with AZD6244, BEZ235, PLX4720, AZD8055, or the indicated combinations, and then assessed by western blot for inhibition of relevant signaling molecules. Drug doses for Me13 and Me6 were: AZD6244: 0.1 μM; BEZ235: 0.1 μM; PLX4720: 0.5 μM; AZD8055: 0.1 μM. Drug doses for Me79 were 0.5 μM for all inhibitors.

**Figure 6:**
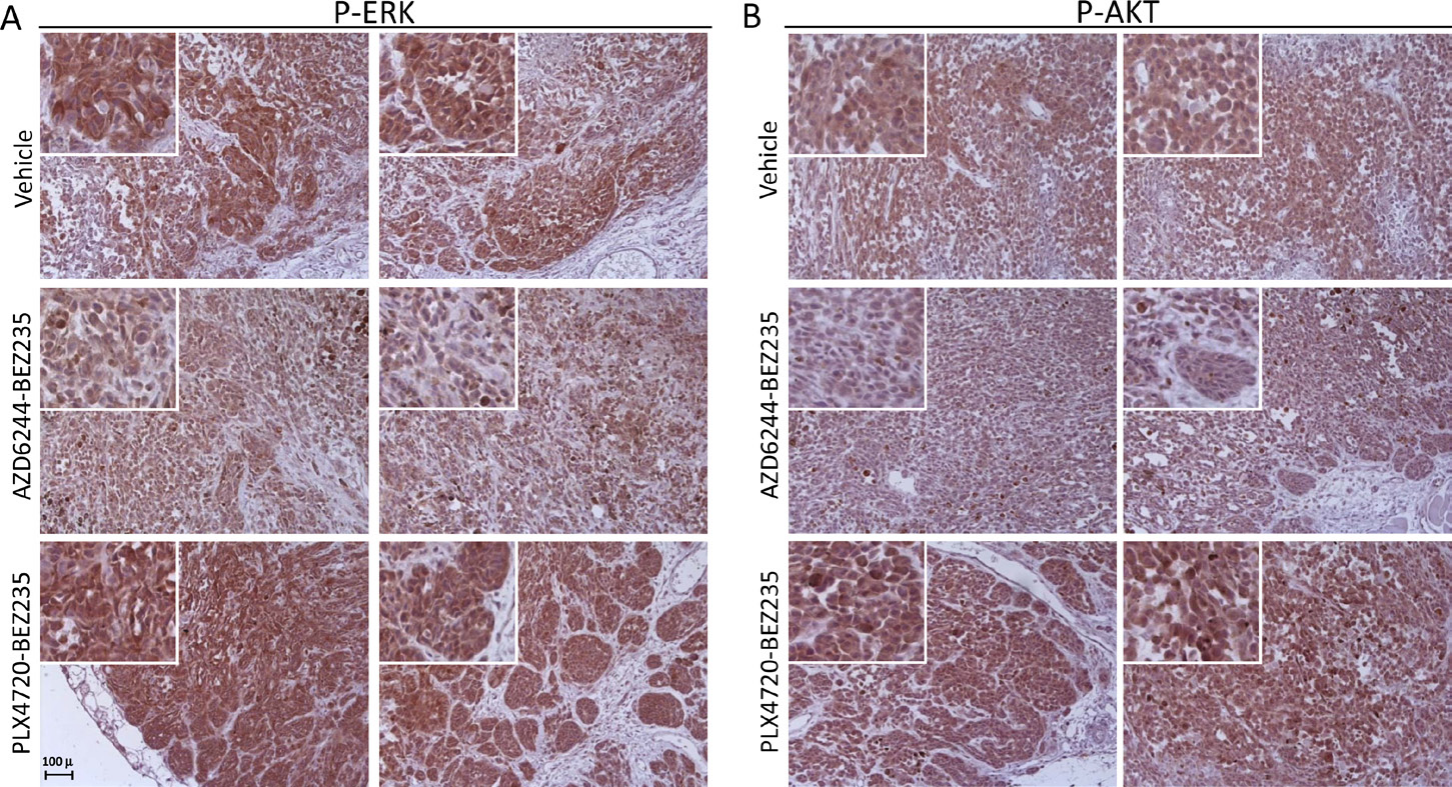
Inhibition of p-ERK and p-AKT *in-vivo* by combinatorial treatments in a PLX4720-resistant cell line Immunohistochemistry analysis by staining with anti-p-ERK (**A**) and anti-pAKT (**B**) antibodies of tumor nodules (images of nodules from two animals are shown for each signaling molecule) removed after the last administration of inhibitors (day 31), from control mice (vehicle) and from mice treated with the association of AZD6244-BEZ235 or of PLX4720-BEZ235, as described in the legend to Supplementary Figure 5. Insets, higher magnification of a representative area of each panel highlighting the extent of staining for p-ERK and p-AKT in melanoma cells. Original magnification, 20x.

Changes in the phosphorylation status of ERK, AKT and S6 by the combinatorial treatments were assessed in three cell lines from group 1 (Me13, Me6 and Me79). Me13 and Me6 have a higher IC_50_ for PLX4720 than for AZD6244, therefore PLX4720 was used at a higher dose compared to AZD6244 (*see legend to* Figure 5). In these normalized conditions, the AZD6244-BEZ235 and AZD6244-AZD8055 associations were more effective than PLX4720-BEZ235 at inhibiting p-ERK in Me13 after O/N treatment, and AZD6244-BEZ235 was the most effective treatment as suppressing p-ERK in Me6 (Figure 5). AZD6244-BEZ235 was also the most effective treatment at inhibiting p-AKT in Me13 and Me6 (Figure 5). All three treatments showed similar inhibitory activity on p-S6 (Figure 5). Enhanced p-ERK inhibition by AZD6244-BEZ235 and AZD6244-AZD8055 compared to PLX4720-BEZ235 were observed also on Me79, a strongly cross-resistant cell line in group1 (Figure 5) as well as on Me71, a cell line from group 3 susceptible to all inhibitors (Supplementary Figure 6). The enhanced suppression of p-ERK by AZD6244-BEZ235 and AZD6244-AZD8055 treatments, compared to PLX4720-BEZ235, was confirmed even after a shorter (4 h) treatment on melanoma cell lines from group 1 (Me13 and Me79, Supplementary Figure 7). At 4 h of treatment, AZD6244-BEZ235 and PLX4720-BEZ235 were effective in suppressing p-AKT on Me13 and Me79, while inhibition of pS6 by the combinatorial treatments was strongly reduced on Me13 and not observed on Me79 compared to the effects detected on the same cell lines after O/N treatment (see Supplementary Figure 7 vs. Figure 5). Enhanced inhibition of p-ERK and p-AKT by AZD6244-BEZ235 compared to PLX4720-BEZ235, and to control animals treated with vehicle, was also observed *in-vivo*, as indicated by a reduced staining for p-ERK and p-AKT in melanoma cells from neoplastic nodules removed after the last administration of inhibitors (Figure 6A, 6B and Supplementary Figure 8 for quantitative analysis).

Taken together, these results indicated that the association of MEK1/2 and PI3K/mTOR inhibitors induces a more effective inhibition of p-ERK at both early and late time points compared to BRAF and PI3K/mTOR dual blockade, in PLX4720-resistant melanoma cells and even in cell lines with a cross-resistant phenotype.

### Enhanced apoptotic response in melanoma cells with a cross-resistant phenotype by co-targeting of MEK1/2 and PI3K/mTOR

**Figure 7:**
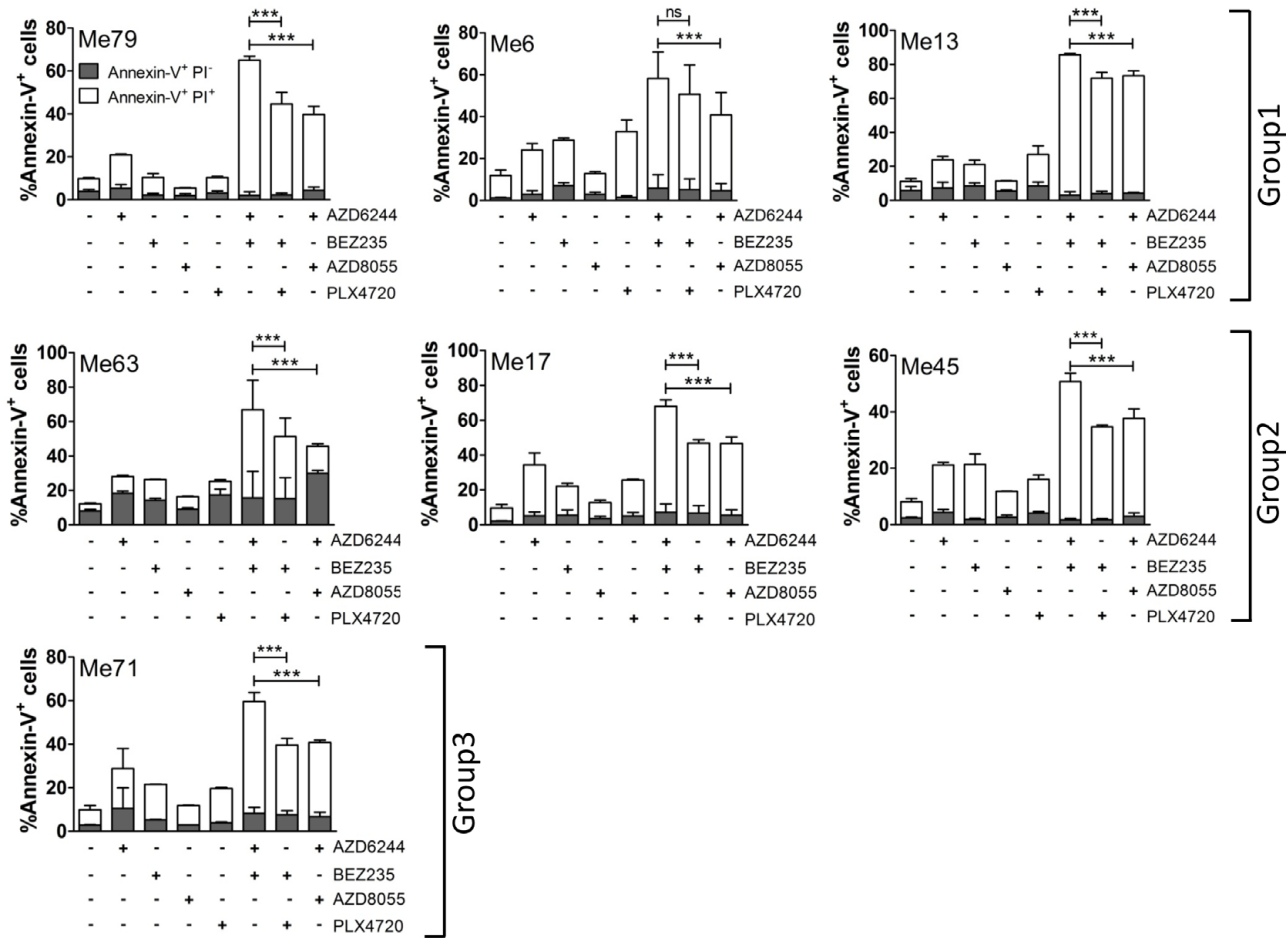
Combinatorial treatments promote melanoma apoptosis Seven melanoma cell lines (representative of the three susceptibility groups) were treated with AZD6244, BEZ235, PLX4720, AZD8055 or the indicated combinations of inhibitors for 72 h, and then apoptosis was assessed by Annexin-V/PI assay. Each histogram is the sum of early (annexin-V^+^/PI^−^, grey) and late (annexin-V^+^/PI^+^, white) apoptosis values. Extent of primary necrosis (% Annexin-V^−^/PI^+^ cells) was always < 5% in control cells and in any of the treatments. Concentrations of inhibitors: Group 1 (Me6 and Me13): AZD6244 0.1 μM, BEZ235 0.1 μM, PLX4720 0.5 μM, AZD8055 0.3 μM; Group 1 (Me79): 0.5 μM for all inhibitors; Group 2 (Me63, Me17, Me45): AZD6244 0.05 μM, BEZ235 0.1 μM, PLX4720 0.1 μM, AZD8055 0.1 μM; Group 3 (Me 71): AZD6244 0.05 μM, BEZ235 0.1 μM, PLX4720 0.1 μM, AZD8055 0.02 μM. Statistical analysis by ANOVA and SNK test. ***p < 0.001.

The most significant effect of AZD6244-BEZ235, compared to PLX4720-BEZ235 and to AZD6244-AZD8055, was a reciprocal shift in the sub-G1 and G1 fractions, while S and G2M phases of the cell cycle were not differentially affected, as documented by DNA content analysis (Supplementary Figure 9) in representative melanoma cell lines from the three PLX4720 susceptibility groups. This result was consistent with enhanced induction of cell death by AZD6244-BEZ235. By annexin-V/PI staining assays we compared extent of apoptosis induced by single inhibitors and by the three combinatorial treatments on representative cell lines from the three groups. To this end, inhibitors doses were tailored to each melanoma group based on the different IC_50_ values (*see legend*, Figure 7). By annexin-V/PI stainings we compared the extent of apoptosis induced by the three combinatorial treatments. AZD6244-BEZ235 was found to be the most effective association, not only against representative cell lines from groups 2 and 3 (Figure 7), but even against melanoma cells with the strong (Me79) or intermediate (Me13) cross-resistant phenotype in group 1 (Figure 7). Further apoptosis assays were carried out by comparing the three combinatorial treatments on all 49 melanoma cell lines. These experiments confirmed that AZD6244-BEZ235 was more effective than PLX4720-BEZ235 against the cell lines in group 1 and group 2 (Supplementary Figure 10A).

**Figure 8:**
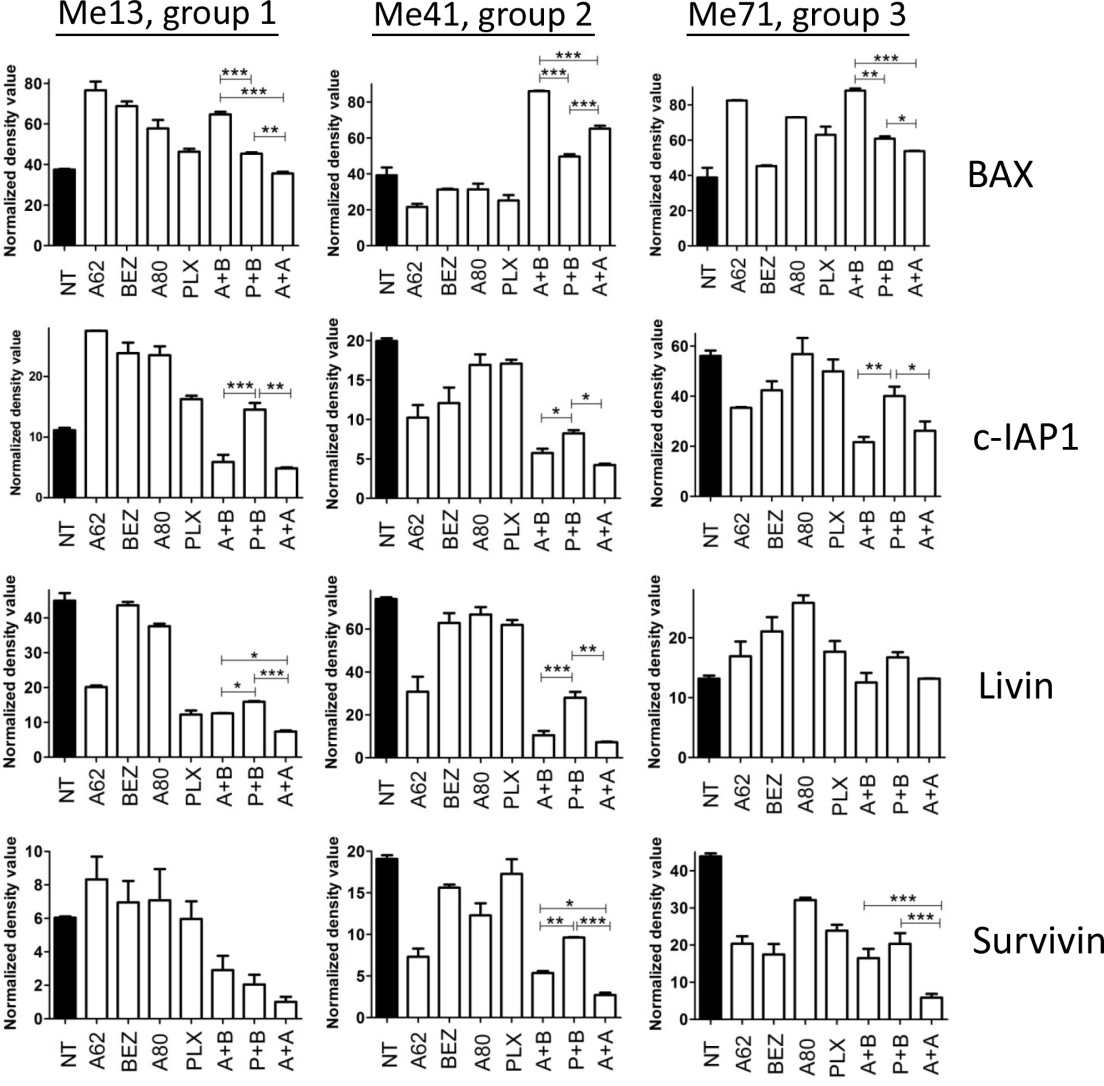
Modulation of pro- and anti-apoptotic proteins by combinatorial treatments Expression of apoptosis-related proteins, by protein array screening, in Me13, Me41 and Me71 cells (representing each of three different susceptibility groups defined in Figure 1) at 48 hours of treatment with or without AZD6244 (A62), BEZ235 (BEZ), PLX4720 (PLX), AZD8055 (A80) and the combinations AZD6244-BEZ235 (A + B), PLX4720-BEZ235 (P + B) and AZD6244-AZD8055 (A + A). Inhibitors concentrations, tailored to the IC_50_ values of the three susceptibility groups, were: Group 1 (Me13): AZD6244 0.1 μM, BEZ235 0.1 μM, PLX4720 0.5 μM, AZD8055 0.3 μM; Group 2 (Me41): AZD6244 0.05 μM, BEZ235 0.1 μM, PLX4720 0.1 μM, AZD8055 0.1 μM; Group 3 (Me 71): AZD6244 0.05 μM, BEZ235 0.1 μM, PLX4720 0.1 μM, AZD8055 0.02 μM. Statistical analysis by ANOVA and SNK test. ***p < 0.001; **p < 0.01; *p < 0.05.

**Figure 9:**
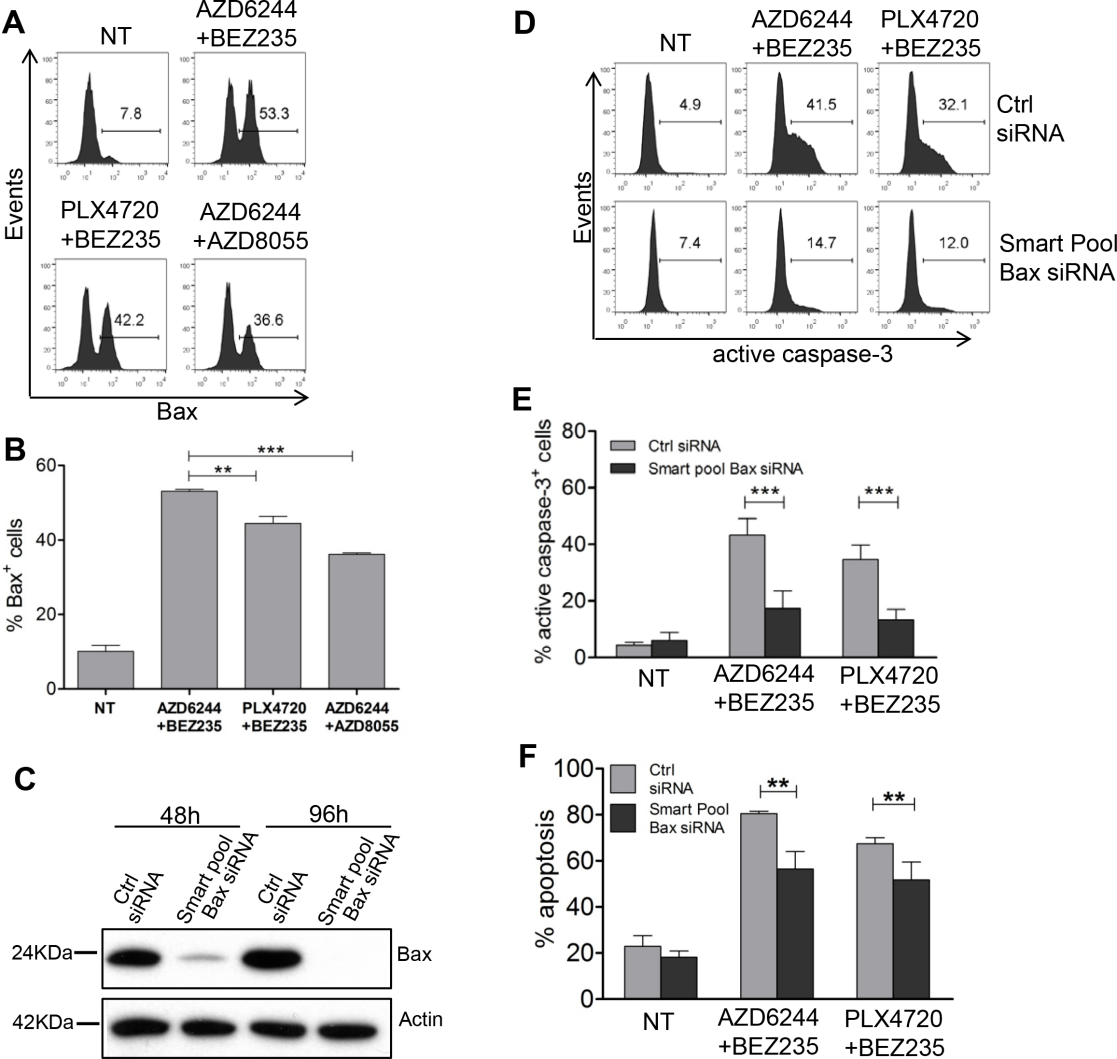
Role of Bax upregulation, by combinatorial treatments, in caspase-3 activation and melanoma apoptosis (**A**, **B**) Me6 melanoma cells were treated for 24 h with the indicated associations of inhibitors and then stained with the Bax conformation-specific mAb 6A7. Inhibitors doses as in Figure 7. (B) Mean of three experiments. (**C**) Western blot analysis for Bax at 48 h and 96 h after transfection with Bax-specific Smart Pool siRNA or with the corresponding negative control siRNA. (**D**–**F**) Me6 cells, transfected with Bax-specific Smart Pool siRNA or with the negative control siRNA, were treated at 48 h with the AZD6244-BEZ235 or PLX4720-BEZ235 associations and analyzed at 96 h for active, cleaved caspase-3 (D, E) or for apoptosis (F). Numbers in each panel in A, D: % positive cells, markers set based on staining with secondary antibody only (A) or isotype control (D). Statistical analysis by ANOVA and SNK test (B) or two-way ANOVA and Bonferroni test (E, F). **p < 0.01, ***p < 0.001.

By protein arrays experiments we found that AZD6244-BEZ235 was more effective than PLX4720-BEZ235 at enhancing expression of the Bax protein, a pro-apoptotic Bcl-2 family member, and at downregulating the inhibitor of apoptosis c-IAP1 in melanoma cells from the three groups and of livin/ML-IAP in melanoma cells from group 1 and 2 (Figure 8). By staining Me6 melanoma cells with the 6A7 antibody, recognizing conformational changes of Bax, one of the early steps in the induction of apoptosis, we found a significantly increased fraction of Bax^+^ melanoma cells upon treatment for 24 h with AZD6344-BEZ235 compared to PLX4720-BEZ235 and to AZD6244-AZD8055 (Figure 9A, 9B). Silencing of Bax in Me6, by siRNA (Figure 9C), significantly reduced activation of caspase-3 and (Figure 9D, 9E) and apoptosis (Figure 9F) upon treatment with AZD6344-BEZ235 or PLX4720-BEZ235.

On the basis of these results, we then tested whether co-targeting of MEK1/2 and PI3K/mTOR could lead to enhanced activation of effector caspases [25] on most cell lines, compared to the other associations. In agreement, AZD6244-BEZ235 combination was more effective than PLX4720-BEZ235 and AZD6244-AZD8055 associations in inducing caspase 3/7 enzymatic activation in assays carried out on all 49 melanoma cell lines (Supplementary Figure 10B). Further assays looking at the generation of active, cleaved caspase-3, indicated that AZD6244-BEZ235 was the most effective treatment, compared to PLX4720-BEZ235 and to AZD6244-AZD8055 in melanoma cells from group 3 (Me71) and from group 1 (Me6, Me13 and Me79, Supplementary Figure 11A, 11B). Assessment of neoplastic nodules removed after the last administration of inhibitors from mice receiving the combinatorial treatments indicated an enhanced activation of caspase-3, associated with induction of apoptosis (TUNEL^+^ cells), in tumors from mice treated with AZD6244-BEZ235 compared to PLX4720-BEZ25 (Supplementary Figure 12).

*In-vitro*, a pan-caspase inhibitor z-VAD-fmk, but not the negative control z-FA-fmk, significantly reduced Me6 cells apoptosis promoted by the combinatorial treatments, indicating that melanoma apoptosis was caspase-dependent (Supplementary Figure 13A, 13B).

Taken together, these results suggest that co-targeting of MEK1/2 and PI3K/mTOR, compared to BRAF and PI3K/mTOR dual blockade, is a more effective approach to rescue susceptibility to caspase-dependent apoptosis in melanoma cells with intrinsic cross-resistant phenotype.

### Selective modulation of genes in the ERK/MAPK and PI3K/AKT canonical pathways by MEK1/2 and PI3K/mTOR dual blockade compared to BRAF and PI3K/mTOR co-targeting

Whole genome gene expression analysis was carried out in the PLX4720-resistant Me13 cell line treated with AZD6244-BEZ235, or PLX4720-BEZ235 or AZD6244-AZD8055 associations, to identify combination-specific effects. To improve the statistical analysis of the results, three independent biological replicates for each treatment were analyzed. Moreover, drug doses were chosen according to the different IC_50_ values; thus, PLX4720 was used at 0.5 μM while AZD6244 was used at 0.1 μM (see Materials and methods for details). Significantly modulated genes by each combination, identified by class comparison through BRB Array Tools, were subjected to downstream effect analysis through IPA software. “Cell death and survival” was the top biological function affected by the three combinatorial treatments, but the most significant *P* value for association with such function was observed for the AZD6244-BEZ235 treatment (data not shown). Based on Z scores > 2 or < −2, the function “cell death” was predicted to be increased, while the function “proliferation” was predicted to be decreased by the three combinatorial treatments (see Supplementary Table 4 for results on AZD6244-BEZ235 treatment). By Edwards-VENN diagram analysis [26] combination-specific gene expression changes were identified (Supplementary Figure 14,*underlined values*) in addition to gene expression changes shared by two or even three different combinatorial treatments (Supplementary Figure 14, *boxed values*). Thus, AZD6244-BEZ235 upregulated a set of 79 genes and downregulated a different set of 83 genes, not significantly affected by the other two combinatorial treatments. By canonical pathway analysis by IPA (Supplementary Figure 15), the specificity of each combinatorial treatment was revealed by two parameters: a) the different ranking of each of the top 15 pathways (based on the *P* value of the association of the genes with the pathway) and, b) the differences, among the three combinatorial treatments, in the fraction of significantly modulated genes belonging to each pathway (*plotted as stacked bar charts in* Supplementary Figure 15). Thus, upon combinatorial treatment with AZD6244-BEZ235, the two top canonical pathways affected were the “PI3K/AKT signaling”, and the “PTEN signaling”. In contrast, upon melanoma treatment with PLX4720-BEZ235 or with AZD6244-AZD8055 these two pathways ranked third and second, or 15th and sixth, respectively (Supplementary Figure 15).

**Figure 10:**
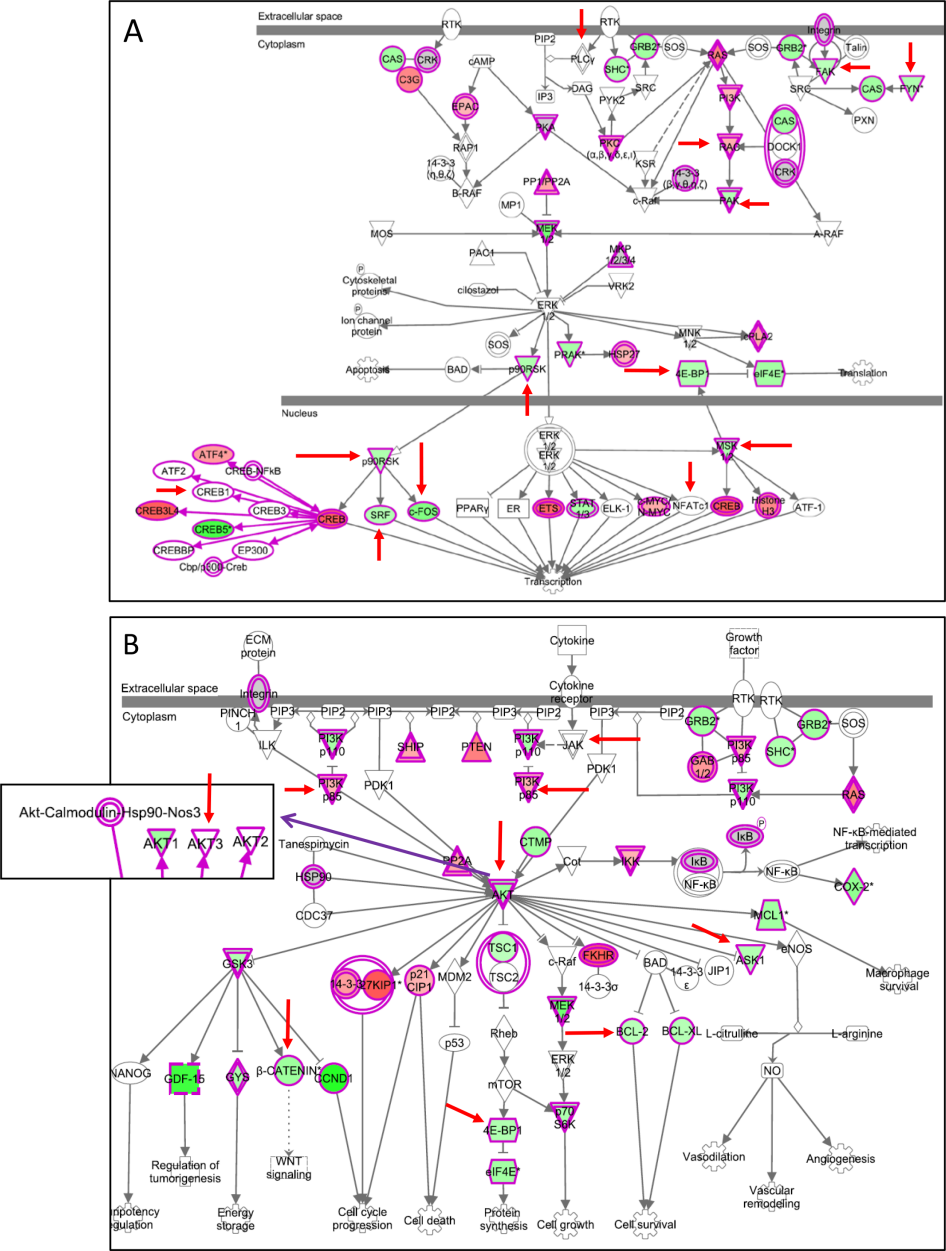
Selective modulation of genes in the ERK/MAPK and PI3K/AKT canonical pathways by AZD6244-BEZ235 (**A**, **B**) genes modulated in Me13 cells by AZD6244-BEZ235 treatment and belonging to the ERK/MAPK canonical pathway (A) and the PI3K/AKT canonical pathway (B). Upregulated genes are shown in red, and downregulated genes in green. Red arrows: genes differently affected by AZD6244-BEZ235 compared to PLX4720-BEZ235 and to AZD6244-AZD8055 treatments (see Supplementary Figure 16 for genes modulated by the latter two treatments).

By looking at genes belonging to three canonical pathways (ERK/MAPK, PI3K/AKT, and “prostate cancer signaling”) specific differences in the modulatory effects induced by the three combinatorial treatments were identified (*highlighted by red arrows in* Figure 10 and Supplementary Figure 16A–16C). This comparison indicated that AZD6244-BEZ235 induced a selective downmodulation of several genes, including c-FOS (Figure 10A), recently involved in melanoma resistance to MAPK inhibition [13], and p90RSK encoding for proteins that phosphorylate CREB transcription factors [27–28], also involved in melanoma resistance to MAPK inhibition [13]. The preferential downmodulation of c-FOS by AZD6244-BEZ235, compared to the effects of the other associations of inhibitors, was confirmed by qPCR (Supplementary Figure 17A). AZD6244-BEZ235, but not PLX4720-BEZ235, downmodulated the anti-apoptotic gene Bcl-2 and the mTOR interactor 4EBP1 (Figure 10B), as well as β catenin, a gene that impairs T cell-mediated immune response in melanoma [29], and LEF-1 [30] a β catenin interacting partner (Supplementary Figure 16B, 16C). The latter two genes are upstream of cyclin D1, a gene being more strongly inhibited (FC = −3.85) by AZD6244-BEZ235, compared to PLX4720-BEZ235 (FC = −2.17). β catenin downmodulation by AZD6244-BEZ235 treatment was confirmed by qPCR (data not shown). By immunohistochemistry in neoplastic nodules removed after the last administration of inhibitors, a reduced staining for β catenin was observed in melanoma cells from animals treated with AZD6244-BEZ235 compared to animals receiving vehicle or the PLX4720-BEZ235 combination (Supplementary Figure 17B).

Collectively, this evidence indicates that the AZD6244-BEZ235 association has an enhanced effect on the biological function “cell death and survival” and a selective modulatory effect on genes that play a role in melanoma resistance to target-specific inhibitors and in suppression of anti-tumor immunity.

## DISCUSSION

Intrinsic resistance to BRAF inhibitors prevents ∼20% of melanoma patients with BRAF-mutant tumors from achieving clinical benefit from this type of target therapy and represents a major clinical issue [31, *for review*]. In this subset of patients, combination treatments based on co-targeting of different oncogenic pathways have been suggested as a potentially effective approaches [32], but the best combinatorial association has not been identified yet. Clinical studies of combinatorial treatment with MEK and PI3K/mTOR inhibitors are ongoing in different solid tumors, including melanoma (source: www.clinicaltrials.gov) and results of published trials [33] suggest that dual targeting of these pathways has clinical activity, although toxicity issues have been identified. Moreover, pre-clinical studies, in different tumors suggest that co-targeting of MAPK and PI3K/mTOR pathways may be a potentially promising strategy, as shown by results obtained in gefitinib-resistant NSCLC cells [34], in pancreatic cancer cells [35], in rhabdomyosarcoma cells [36], in NRAS mutant melanoma cells [17] and in BRAF mutant melanoma cells with acquired resistance mediated by upregulation of PDGFRβ [16].

In this study we provide novel preclinical evidence indicating that primary resistance to BRAF inhibition is not only frequently associated with cross-resistance to MEK1/2 inhibitors, but even with cross-resistance to PI3K/mTOR inhibitors. Based on this evidence, we tested whether a combinatorial treatment approach could be effective in the subset of melanoma cells with the intrinsic cross-resistant phenotype. The results indicated that the most significant synergistic effects (as determined by combination index values) could be achieved by co-targeting of MEK1/2 and PI3K/mTOR. Co-targeting of MEK1/2 and PI3K/mTOR was significantly more effective than BRAF and PI3K/mTOR dual blockade not only *in-vitro*, but also *in-vivo*, against xenografts from a cell line with intrinsic resistance to the BRAF inhibitor PLX4720. The anti-melanoma efficacy of the AZD6244-BEZ235 treatment was associated with strong inhibition of key signaling molecules, as documented by modulation of p-ERK and of p-AKT, both *in-vitro* and *in-vivo*. Interestingly, in melanoma cells with acquired resistance to BRAF inhibition, Shi et al. [16] found that PLX4720, alone or in combination with BEZ235 or AZD8055, induced early and delayed p-ERK recovery, while AZD6244 in association with BEZ235 or AZD8055, strongly reduced such effect.

Assessment of the mechanism(s) involved in the enhanced anti-melanoma efficacy by co-targeting of MEK1/2 and PI3K/mTOR, compared to mutant BRAF and PI3K/mTOR dual inhibition, provided evidence for a more effective induction of apoptosis, not only *in-vitro*, but also *in-vivo* and even in cross-resistant cell lines. Treatment by AZD6244-BEZ235 was associated with enhanced modulation of pro- and anti-apoptotic molecules, compared to PLX4720-BEZ235 and to AZD6244-AZD8055. Further assessment of the mechanism of melanoma apoptosis, after dual pathway co-targeting, indicated that AZD6244-BEZ235 was the most effective combination at inducing activation of Bax and of caspase-3, the latter effect being observed both *in-vitro* and *in-vivo*. Silencing of Bax reduced both caspase-3 activation and melanoma apoptosis. Further assays with a pan-caspase inhibitor also indicated that melanoma apoptosis was significantly inhibited by a pan-caspase inhibitor, suggesting that one of the relevant effects of the combinatorial treatments is to rescue susceptibility of melanoma to caspase-dependent cell death.

Analysis for combination-specific effects on gene expression, provided evidence for selective modulation of genes belonging to the ERK/MAPK and PI3K/AKT canonical pathways by the AZD6244-BEZ235, compared to PLX4720-BEZ235 and to AZD6244-AZD8055. The inhibitory effects on c-FOS, and on genes (p90RSK) encoding proteins that affect CREB phosphorylation, suggest that AZD6244-BEZ235 is a potentially effective approach to overcome recently described mechanisms mediating melanoma resistance to MAPK inhibition [13]. In agreement with this interpretation, caspase 3/7 activation assays and cell death assays indicated that AZD6244-BEZ235 was significantly more effective in the promotion of apoptosis on melanoma cell lines of all susceptibility groups. Thus, these results support the notion that rescuing susceptibility to apoptosis is a major mechanism of action of effective co-targeting strategies in melanoma, as reported previously by us [18, 37–38], and other groups in melanoma [16] and other tumors [35].

The selective downmodulation of β catenin, by AZD6244-BEZ235 compared to PLX4720-BEZ235, suggests that dual blockade of the MAPK and PI3K/mTOR pathways could suppress a recently discovered mechanism that melanoma cells exploit to suppress development of T cell mediated anti-tumor response [29] and provides a further rationale for the association of target therapy, based on dual pathway inhibition, with immune checkpoint blockade, an approach that has greatly improved the management of advanced disease [39].

Analysis of responsiveness to BRAF, MEK1/2 and PI3K/mTOR inhibitors in a few short term melanoma cell cultures, from patients subsequently treated with target therapy, suggested that drug susceptibility data may predict response or resistance to treatment. Clearly, this hypothesis needs confirmation in a larger set of patients, but the testing of freshly isolated melanoma cells from surgical samples, for responsiveness to available inhibitors, is feasible in principle in advanced melanoma and could provide a valuable evidence to inform subsequent clinical decisions.

## MATERIALS AND METHODS

### Ethics statement

This investigation has been conducted in accordance with the ethical standards and according to the Declaration of Helsinki and according to national and international guidelines and has been approved by the independent ethical committee of our Institute. *In-vivo* experiments in SCID mice were performed according to the Italian laws (D.L. 116/92 and after additions), after approval by the institutional Ethical Committee for Animal Experimentation of our Institute and by the Italian Ministry of Health (Project INT_17/2011).

### Cell lines and short-term melanoma cell cultures

Forty-nine BRAF-mutant melanoma cell lines were established as described [18, 37–38, 40–41] from surgical specimens of American Joint Committee on Cancer (AJCC) stage IIIc and IV melanoma patients not previously subjected to target therapy. An independent panel of 33 short-term melanoma cell cultures was generated as described [40–41] from surgical samples of AJCC stage IIIc and IV BRAF mutant (*n* = 23) or wild type (*n* = 10) patients not previously subjected to target therapy. Short-term melanoma cell cultures were used between the third and fifth *in-vitro* passage. All patients were admitted to Fondazione IRCCS Istituto Nazionale dei Tumori, Milan and all the lesions were histologically confirmed to be cutaneous malignant melanomas. Informed consent was obtained from patients. Molecular and biological characterization of the cell lines and methods for identification of mutations in BRAF gene have been reported previously [18, 37–38, 41–42].

### Treatment of melanoma cells with inhibitors and drug interaction analysis

Two days before treatment, melanoma cells were seeded in 96-wells flat bottom plates in RPMI 1640 (BioWhittaker) supplemented with 2% fetal calf serum (FCS) without antibiotics. For IC_50_ determination, treatments were in quadruplicate with AZD6244 (MEK1/2 inhibitor, SelleckChem), PLX4720 (BRAFV600E inhibitor, SelleckChem), BEZ235 (dual PI3K and mTOR inhibitor, SelleckChem), AZD8055 (dual mTORC1/2 inhibitor, SelleckChem). Stocks and dilutions of inhibitors were done in DMSO. Twelve concentrations of each inhibitor ranging from 1 nM to 10 μM at final FCS concentration of 1% were used. Cultures were evaluated at 72 hours as described [18, 37–38] by the 3-(4, 5) dimethylthiazol-2, 5-diphenyltetrazolium bromide (MTT) assay. IC_50_ values were obtained through nonlinear regression analysis (by PRISM software, Graphpad) of dose-response curves by a log (inhibitor) vs. response, variable slope equation. Clonogenic assays were performed by seeding melanoma cells at single-cell density in 6-well plates. Treatments with AZD6244, BEZ235, PLX4720, and AZD8055 (0.1–0.5 μM), either alone or in combination, vs. DMSO were done every 72 h. After 12 days, the supernatant was discarded, plates were washed with HBSS, fixed with methanol and then stained with Giemsa (Sigma Aldrich) followed by Image J quantification of images. Results of clonogenic assays were expressed as % inhibition of melanoma growth. For drug interaction analysis the Chou and Talalay method was used [24]. To this end, MTT assays were set up testing three combinations of inhibitors (AZD6244-BEZ235, PLX4720-BEZ235 and AZD6244-AZD8055). For each combinatorial treatment, twelve different combinations of doses were used. Data were then analyzed to obtain Combination index (CI) and Fraction Affected (FA) values by the CompuSyn software (ComboSyn).

### Antibodies and western blot analysis

Western blot analysis was carried out by the following antibodies specific for: AKT, p-AKT (Ser473), ERK 1/2, p-ERK1/2 (Thr202/Tyr204), S6, p-S6 (Ser235/236), PTEN and Bax (Cell Signaling); MDM4 (Bethyl Laboratories), MDM2 (Santa Cruz), β-actin (Sigma-Aldrich), α-tubulin (Calbiochem) and Vinculin (Sigma-Aldrich). SDS-PAGE was performed using 30 μg of protein samples on 4%–12% NuPAGE Bis-Tris polyacrylamide gels (Invitrogen Life Technologies), as described [18, 37–38]. Development was performed by the chemiluminescence method with the ECL Western Blotting Detection System (GE Healthcare) or Luminata Crescendo (Millipore). Basal/constitutive phosphoprotein characterization was carried out after O/N culture without FCS. Modulation of expression of phosphoproteins, by inhibitors, was assessed in melanoma cells treated either for 4 hr or O/N with MAPK and PI3K/mTOR pathways inhibitors, alone or in combinations, with 2% FCS. Protein quantification was performed by densitometric analysis with the Quantity One software (BioRad Laboratories).

### *In-vivo* evaluation of anti-melanoma activity of combinatorial treatments

Female SCID mice, 8–10 weeks old (Charles River Laboratories) were provided with food and water *ad libitum*. Melanoma cells (Me13), harvested in exponential growth phase, were injected s.c. (5×10^6^) in the left flank of each mouse. When tumors became palpable, mice were randomized into three groups (7 animals/group) and animals received either vehicle, the association of AZD6244 (10 mg/kg) and BEZ235 (20 mg/Kg) or of PLX4720 (10 mg/Kg) and BEZ235 (20 mg/Kg), 5 days per week for three consecutive weeks by oral gavage. Mice were monitored daily for signs of toxicity and were weighed twice weekly. Tumor size was regularly evaluated by measuring the orthogonal diameters (d and D) and calculating the volumes with the following formula: 4/3π [(d^2^D)/2].

### Flow cytometry analysis

Melanoma apoptosis was assessed after staining with APC-conjugated Annexin V (BD Pharmingen) and propidium iodide (PI; BD Biosciences) as described [37–38]. DNA content analysis was carried out after staining with propidium iodide (Sigma-Aldrich) as described [37]. Staining with purified mouse Bax conformation-specific antibody 6A7 or with rabbit FITC-anti-active caspase-3 antibody (BD Pharmingen) was carried out on cells permeabilized with CytoFix/CytoPerm (BD Pharmingen). Secondary and control antibodies were, respectively, FITC-labeled goat anti-mouse (Jackson Immunoresearch Laboratories) and FITC-rabbit IgG isotype control. In some experiments, melanoma cells were pre-incubated with general caspase inhibitor z-VAD-fmk or control z-FA-fmk (BD Pharminge) at 5 μM for 1 h at 37°C before treatment with drugs. All experiments were carried out with a FACSCalibur flow cytometer (BD Biosciences) and analyzed by the FlowJo software (Tree Star).

### Cell death and caspase 3/7 activity assays by Muse cell analyzer

In some experiments, apoptosis and caspase 3/7 activation were assessed by the Muse^™^ Cell Analyzer (Merck Millipore). For apoptosis determination, cells were collected and resuspended according to manufacturer's instructions with the working solution of the Muse^™^ Annexin V & Dead Cell Kit (Merck Millipore) for 20 minutes at room temperature. For caspase 3/7 activity cells were collected, resuspended in 1X Assay Buffer BA, and incubated with Muse^™^ Caspase-3/7 Assay Kit (Merck Millipore) reagent working solution for 30 minutes in the 37°C incubator with 5% CO_2_. After incubation, Muse^™^ Caspase 7-AAD working solution was added for 5 minutes at room temperature. Data were analyzed by Muse 1.4 Analysis Software.

### Apoptosis antibody array

Melanoma cells were treated with MAPK and PI3K/mTOR inhibitors for 48 h. The Human Apoptosis Array Kit (R & D Systems) was used, as described [37] according to manufacturer's instructions. The intensity of protein signals was quantified by densitometric analysis with the Quantity One software (BioRad Laboratories). After background subtraction, results were expressed as the percentage of the mean of the relative positive controls.

### Bax silencing experiments

Transient silencing experiments were carried out with ON-TARGET Plus Human BAX siRNA smart-pool (#L-003308–01–0005, Dharmacon GE Healthcare) containing four oligos specific for Bax (#J-003308–11, –12, –13, –14, Dharmacon) or corresponding negative control (#D-001320–10–05, Lincode non targeting pool, Dharmacon). Oligos were used at 10 nm final concentration according to Lipofectamine RNAiMAX guidelines (Thermo Fisher Scientific). Silencing was checked by Western blot analysis at 48 h and at 96 h. At 48 h after transfection with Bax siRNA smart pool, or with negative control oligos, cells were treated with AZD6244 plus BEZ235 (0.1 μM + 0.1 μM), or with PLX4720 plus BEZ235 (0.1 μM + 0.5 μM) and analyzed after 48 h by staining for Annexin-V/Propidium Iodide or for active caspase-3.

### Immunohistochemistry

Immunohistochemistry was performed with formalin-fixed, paraffin-embedded tissues as described [40]. SCID mice bearing s.c. Me13 xenografts were treated with AZD6244-BEZ25 or PLX4720-BEZ235 combinations as described for the tumor growth inhibition assays. Neoplastic nodules were removed after the last administration of inhibitors (day 31) and were characterized by staining with antibodies to p-ERK (Thr202/Tyr204, Cell Signaling), p-AKT (Ser473, Cell Signaling), β catenin (BD Transduction Laboratories), or cleaved caspase-3 (Cell Signaling). The extent of apoptosis in neoplastic nodules was evaluated by TUNEL staining (Roche). Cytospin preparations of melanoma cell lines were processed and stained for AXL (R & D Systems) and for MITF (Dako) as described [43]. Images were acquired at 20x with an Axiovert 100 microscope (Zeiss) equipped with a digital camera (AxioCam MrC5, Zeiss). For image acquisition, all main microscope and digital camera operative settings, including exposure time, were kept constant. Immunohistochemistry images were quantified using ImageJ.

### Genome-wide expression profiling of melanoma cells treated with MAPK- and PI3K/mTOR-specific inhibitors

Melanoma cells from Me13 cell line were treated with AZD6244 (0.1 μM), BEZ235 (0.1 μM), AZD8055 (0.3 μM) or PLX4720 (0.5 μM), or with the AZD6244-BEZ235, PLX4720-BEZ235, AZD6244-AZD8055 combinations for 8 hr. Three biological replicates for each treatment were set up. Total RNA isolation, clean-up, DNase treatment and assessment of RNA integrity and purity were performed as described [18, 38]. Single-color hybridization of RNAs was performed on Illumina Bead Chip HumanHT-12_v4 Microarrays (Illumina). The expression profiles have been deposited in NCBI's Gene Expression Omnibus (GEO) with GSE accession number GSE59882. Background correction, filtering of data, and quantile normalization were done using the BeadStudio Illumina software. Identification of significantly modulated genes was carried out by BRB array tools (vers 4.3.0) developed by Dr. Richard Simon and Amy Peng Lam. Generation of Edwards-VENN diagrams was obtained by VENNTURE software [26]. Downstream effects analysis and canonical pathway analysis were performed by Ingenuity Pathway analysis, IPA 8.5 (www.ingenuity.com) as described [18].

### Real time PCR

c-Fos (Hs99999140-m1), CTNNB1/β catenin (Hs003550489-m1) and, as endogenous control, GAPDH (Hs00266705-g1) TaqMan Gene Expression Assays (Applied Biosystems, Foster City, CA) were used. Total RNA (1 μg) was reverse transcribed with oligo d (t) using a Transcriptor First Strand cDNA Synthesis Kit (Roche, Penzberg, Germany). Preliminary experiments were conducted for the endogenous control using the *C*_t_ slope method to ensure that the quality of each complementary DNA and the dynamic range of amplifications were comparable [44]. Real-time PCR was then carried out with 20 ng input complementary DNA, 1 × TaqMan Gene Expression Master Mix and TaqMan Gene Expression Assays on a ABI PRISM 7900 HT thermal cycler (Applied Biosystems). Data were analyzed using ABI PRISM Sequence Detection Software version 2.2.2 (Applied Biosystems). Relative expression was determined on triplicate reactions using the formula 2^−Δ*Ct*^, reflecting target gene expression normalized to endogenous control levels [44].

### Statistical analysis

IC_50_ values were clustered by Cluster 3.0 software and clustering was visualized by Java TreeView. Correlation of susceptibility profiles to different inhibitors was tested by Spearman correlation analysis. Comparison of IC_50_ values, of combination index (CI) values and of fraction affected (FA) data, in different groups of melanoma cell lines, was done by Kruskal-Wallis and Dunn multiple comparison test. Analysis of FA values by combinations of inhibitors in the same group of melanoma cell lines, or in all cell lines, when equivalent doses of different inhibitors were used, was carried out by Wilcoxon matched pair test. Significance of different treatments on melanoma apoptosis, caspase activation, modulation of apoptosis-related molecules was assessed by ANOVA, followed by Student-Newman-Keul (SNK) multiple comparison test. Analysis of the antitumor activity of different treatments *in-vivo* was carried out by mixed effects model ANOVA [45] by XLSTAT software (Xlstat).

## SUPPLEMENTARY MATERIALS FIGURES AND TABLES










