# Supplementary material for: Primary cross-resistance to BRAFV600E-, MEK1/2- and PI3K/mTOR-specific inhibitors in BRAF-mutant melanoma cells counteracted by dual pathway blockade
Source: Oncotarget. 2015 Dec 14;7(4):3947–65. doi: 10.18632/oncotarget.6600 (PMC4826182; doi:10.18632/oncotarget.6600)
Supplement: Supplementary file 1 [file oncotarget-07-3947-s001.pdf]

Primary cross-resistance to BRAFV600E-, MEK1/2- and PI3K/mTOR-specific inhibitors in BRAF-mutant melanoma cells counteracted by dual pathway blockade

Supplementary Materials

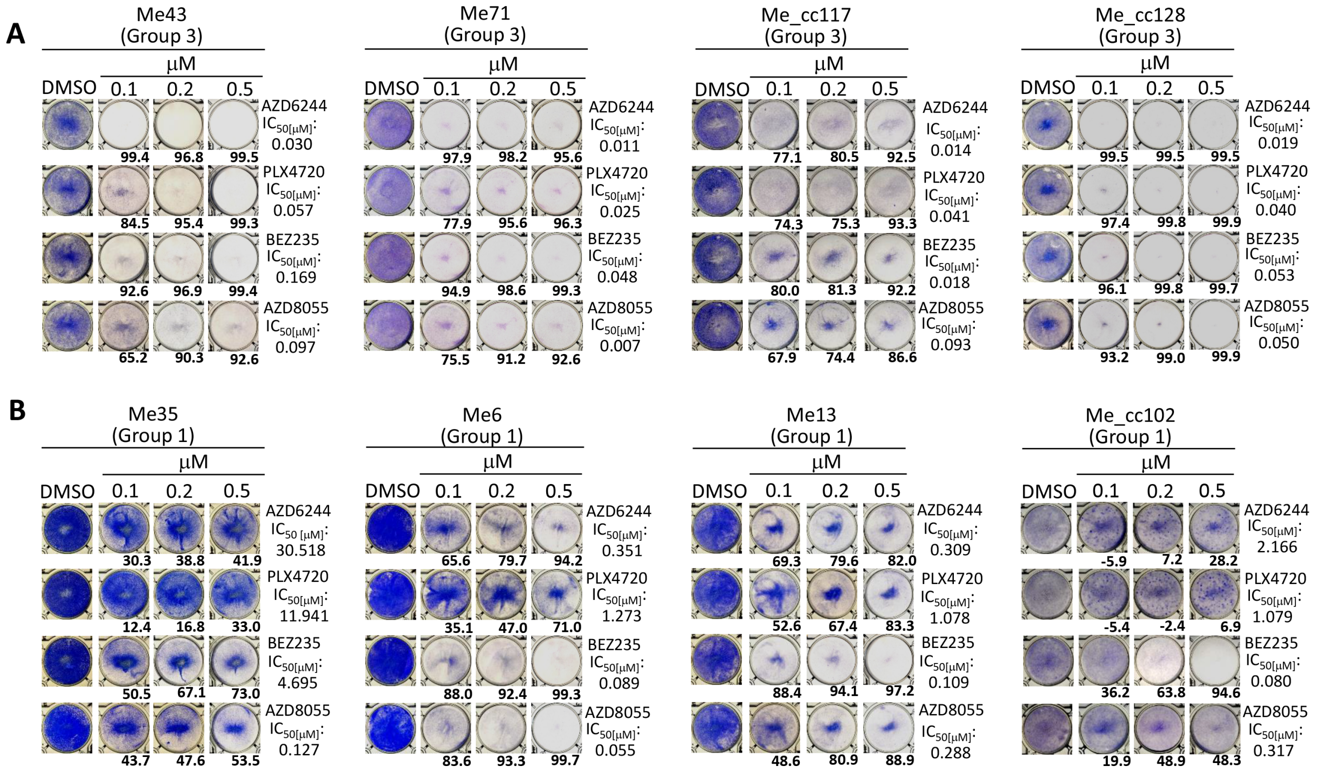

Supplementary Figure S1: Clonogenic assay on melanoma cell lines and on short-term melanoma cell cultures. (**A, B**) Melanoma cell lines (Me43, Me71) and short term melanoma cell cultures (Me_cc117, Me_cc128) from group 3 (A), or group 1 (Me35, Me6, Me13, Me_cc102, (B), seeded in 6-well plates, were treated with AZD6244, PLX4720, BEZ235, or AZD8055 (0.1–0.5 μM) every 72 h. After 12 days, plates were fixed, stained and quantified by Image J. Numbers in the lower right side of each image: % inhibition of melanoma growth relative to DMSO.

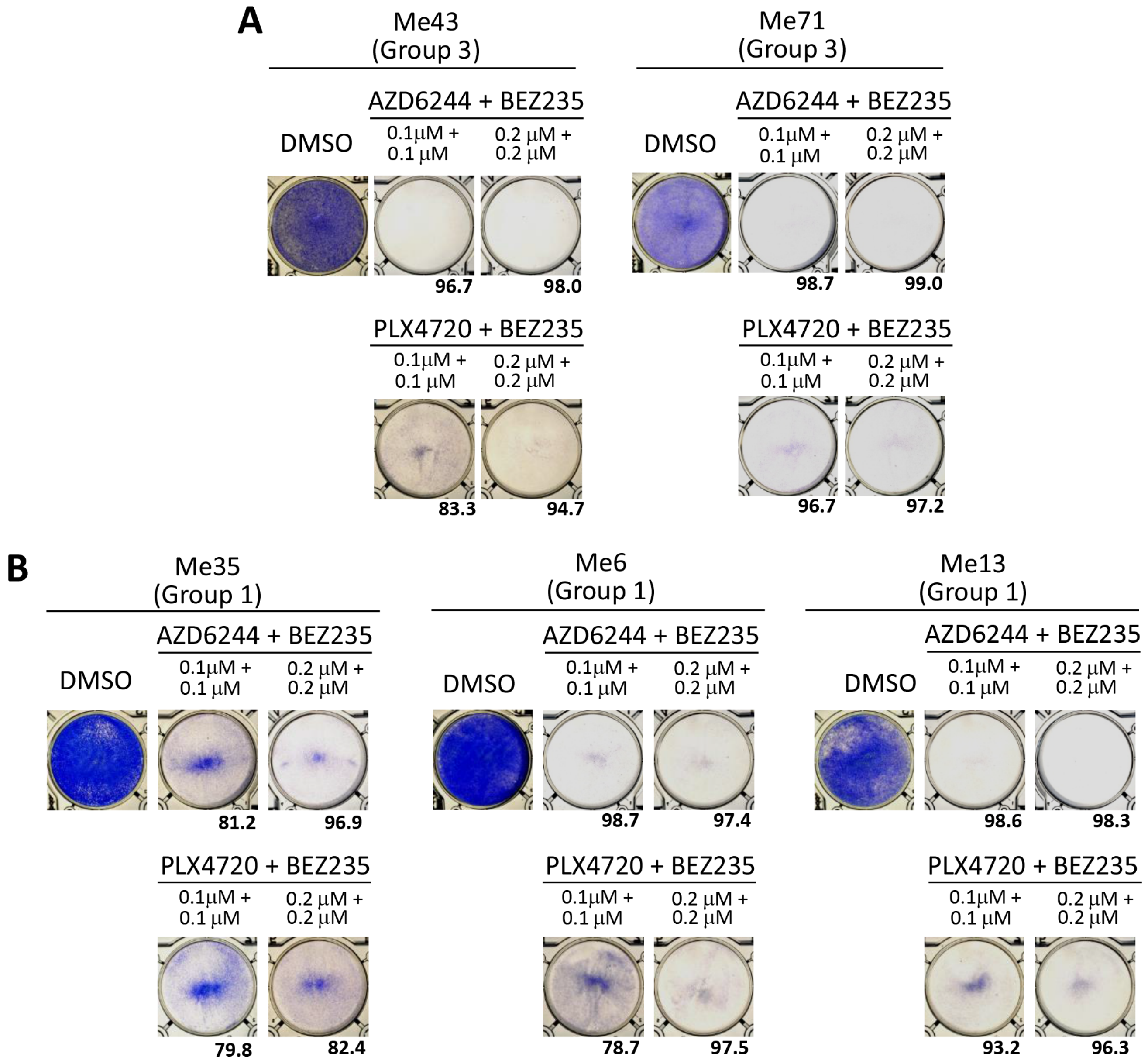

Supplementary Figure S2: Inhibition of clonogenic melanoma growth by combinatorial treatments. (**A**, **B**) Melanoma cells from group 3 (A) or group 1 (B), seeded in 6-well plates, were treated with the association of AZD6244 and BEZ235 or of PLX4720 and BEZ235 at the indicated doses. Cells were treated with the associations of inhibitors every 72 h and evaluated after 12 days as indicated in the legend to Supplementary Figure 1. Numbers in the lower right side of each image: % inhibition of melanoma growth relative to DMSO.

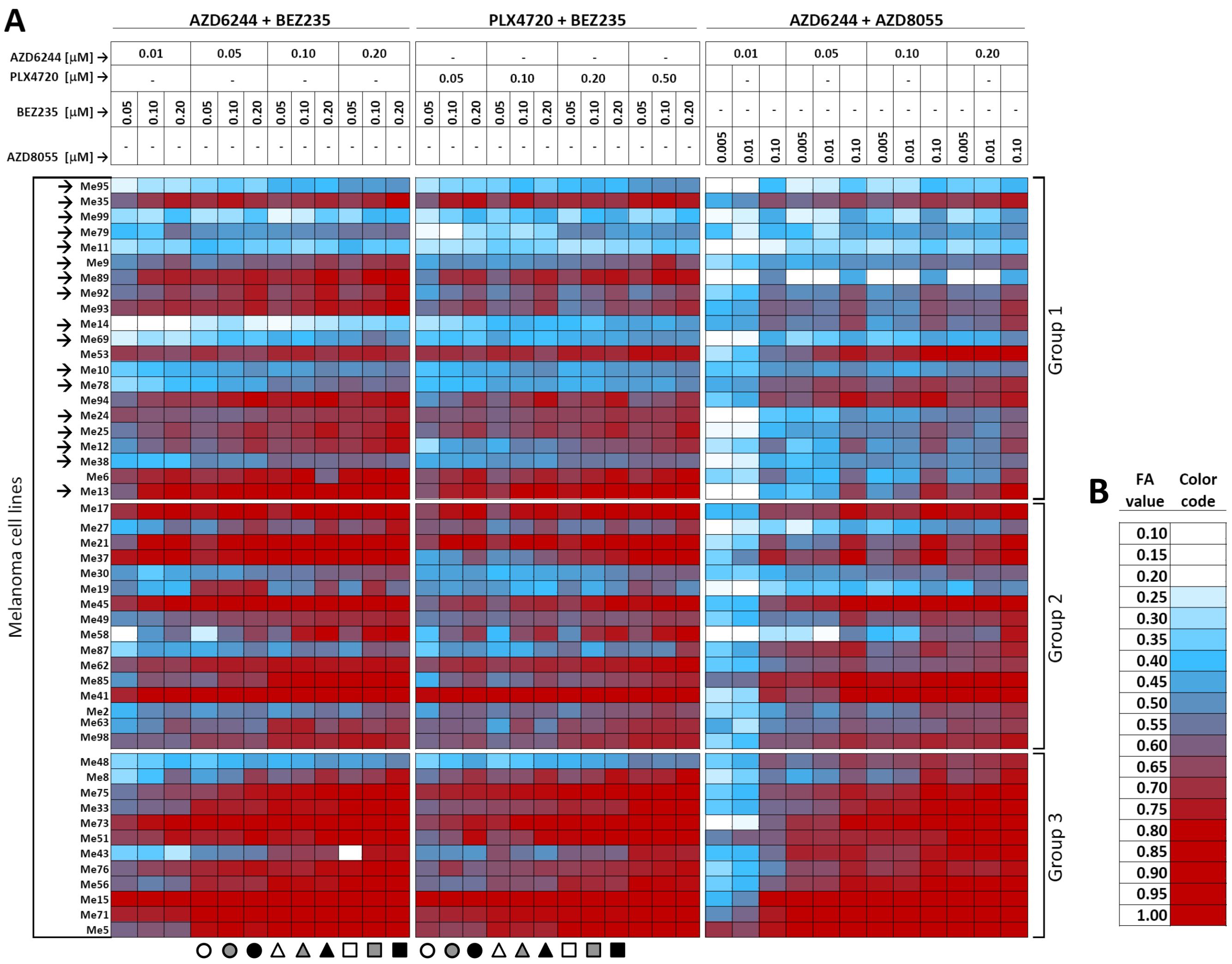

Supplementary Figure S3: Fraction affected (FA) values, by drug interaction analysis, upon combinatorial treatments of melanoma cell lines with different susceptibility profiles. (**A**) Fraction affected values (FA) by combinatorial treatments in the three groups of melanoma cell lines as in Figure 4. Arrows: cell lines (*n* = 17) with a strong cross-resistant phenotype in susceptibility group 1. Matched symbols at the bottom of panel A identify combinations of AZD6244-BEZ235 and PLX4720-BEZ235 where AZD6244 and PLX4720 were used at equivalent doses. (**B**) Color code for FA values.

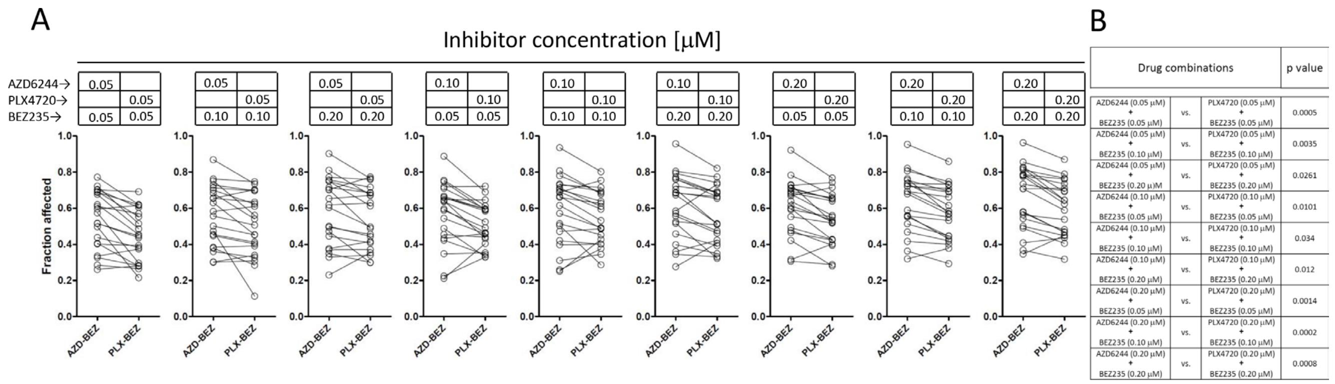

Supplementary Figure S4: Comparison of FA values observed by AZD6244-BEZ235 vs PLX4720-BEZ235 treatments in melanoma cell lines from group 1. (**A**) FA values achieved on 21 melanoma cell lines from group 1 and in the 9 matched drug combinations where AZD6244 and PLX4720 were used at equivalent doses. (**B**) Wilcoxon matched pair test of data shown in (A).

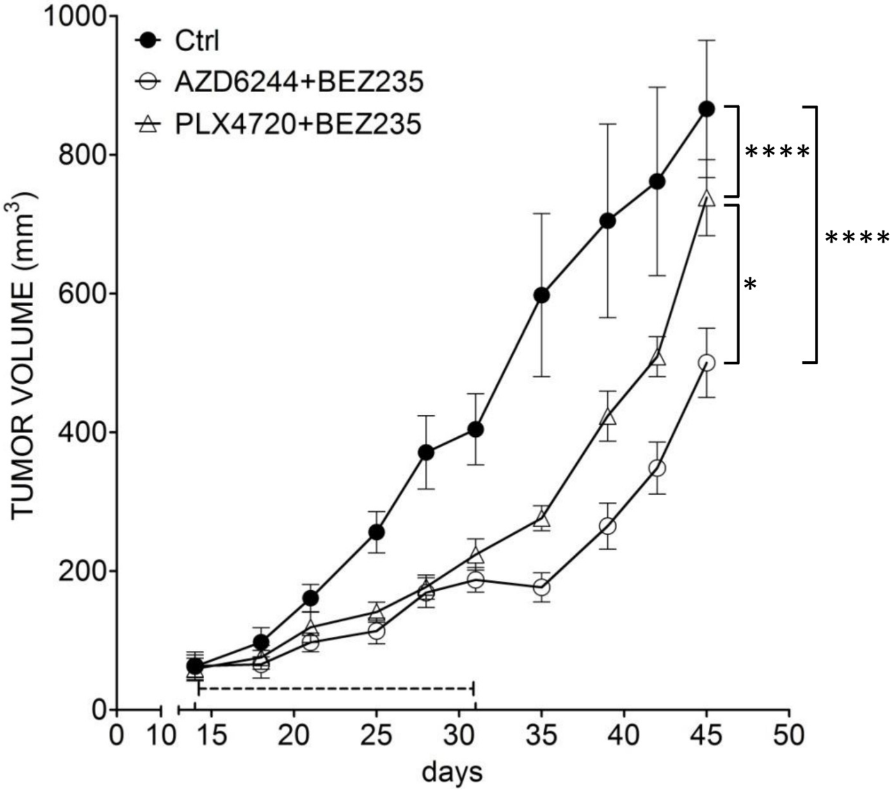

Supplementary Figure S5: Tumor growth inhibition *in vivo*, by combinatorial treatments with AZD6244-BEZ235 *vs* PLX4720-BEZ235. Female SCID mice (n = 7/group) bearing Me13 xenografts were treated (by oral gavage) between day 13 and day 31 (dotted line) with either vehicle (Ctrl), or the association of AZD6244 (10 mg/kg) and BEZ235 (20 mg/Kg) or of PLX4720 (10 mg/Kg) and BEZ235 (20 mg/Kg), 5 days per week for three consecutive weeks. Statistical analysis by mixed models ANOVA; ****p < 0.0001; *p = 0.025.

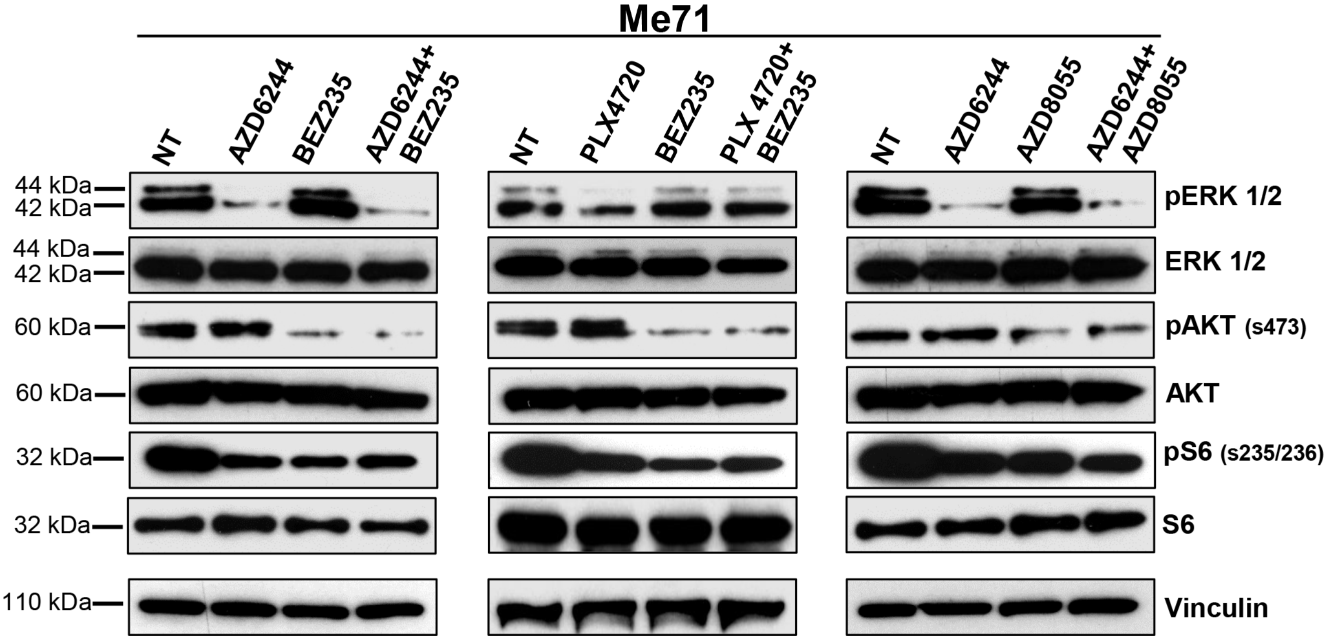

Supplementary Figure S6: Differential inhibition of signaling molecules in the MAPK and PI3K/mTOR pathways by AZD6244-BEZ235 *vs* PLX4720- BEZ235 *vs* AZD6244-AZD8055 combinatorial treatments. A melanoma cell line from group 3 (Me 71, one of the most susceptible to all inhibitors in the whole panel of cell lines) was treated O/N with AZD6244, BEZ235, PLX4720, AZD8055 or the indicated combinations and then assessed by western blot for target inhibition. Me71 was treated with AZD6244 at 0.05 µM, PLX4720 at 0.1 µM, BEZ235 at 0.1 µM and AZD8055 at 0.02 µM.

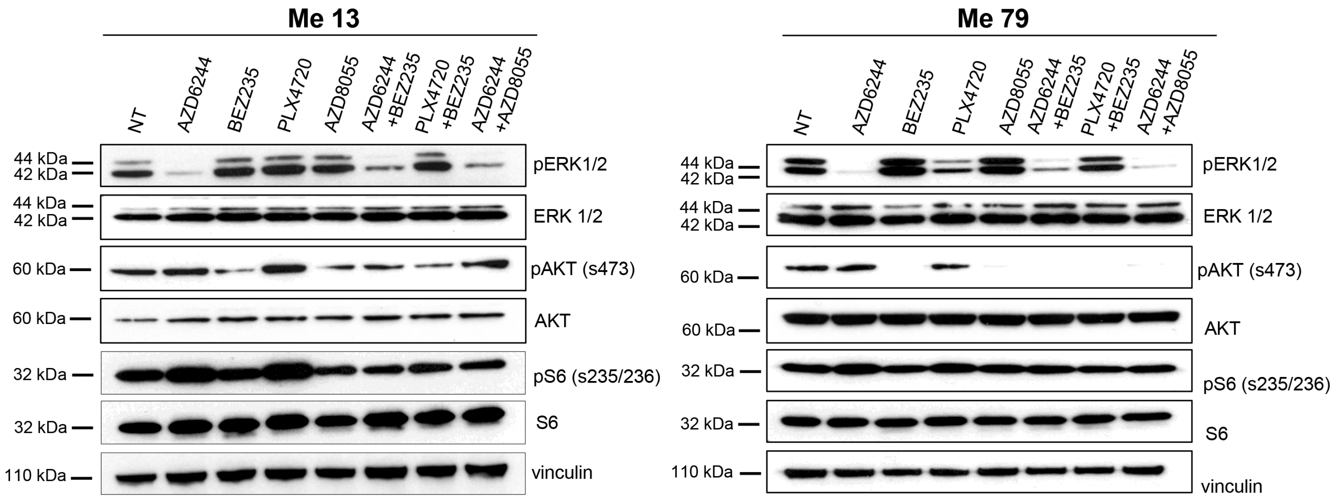

Supplementary Figure S7: Inhibition of p-ERK and p-AKT by combinatorial treatments in melanoma cells with intrinsic resistance to PLX4720. Two melanoma cell lines from group 1 (Me13, Me79) were treated for 4 h with AZD6244, BEZ235, PLX4720, AZD8055, or the indicated combinations, and then assessed by western blot for inhibition of relevant signaling molecules. Drug doses were as in the legend to Figure 5.

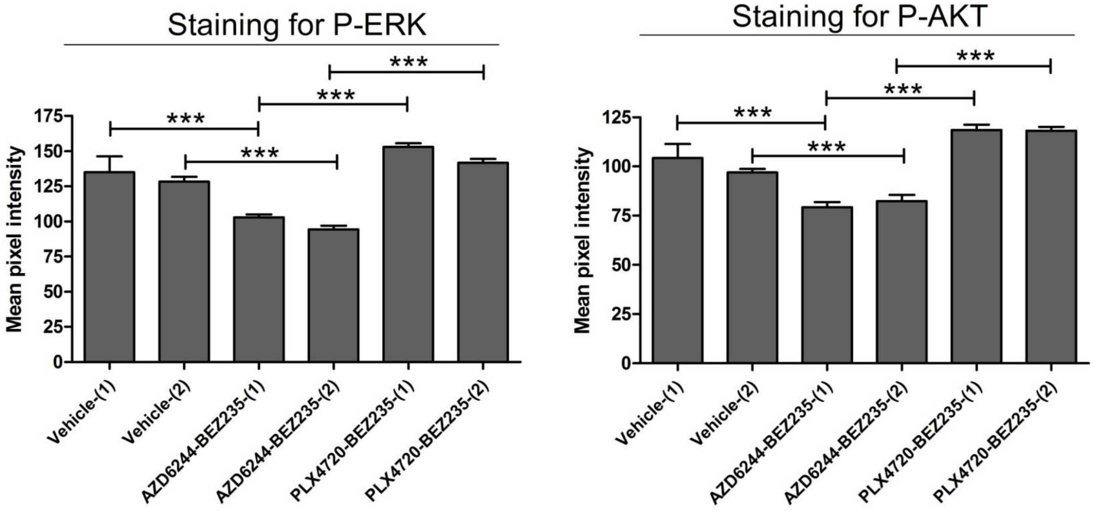

Supplementary Figure S8: Inhibition of p-ERK and p-AKT *in-vivo* by combinatorial treatments in a PLX4720-resistant cell line. Images shown in Figure 6, related to tumor nodules from two animals (1 and 2) treated with vehicle, or with the association of AZD6244-BEZ235 or of PLX4720-BEZ235, and then stained for p-ERK or p-AKT, were analyzed by Image J. For each image, six 800x800 pixel areas were analyzed. Statistical analysis by ANOVA and SNK test. ***$p < 0.001$.

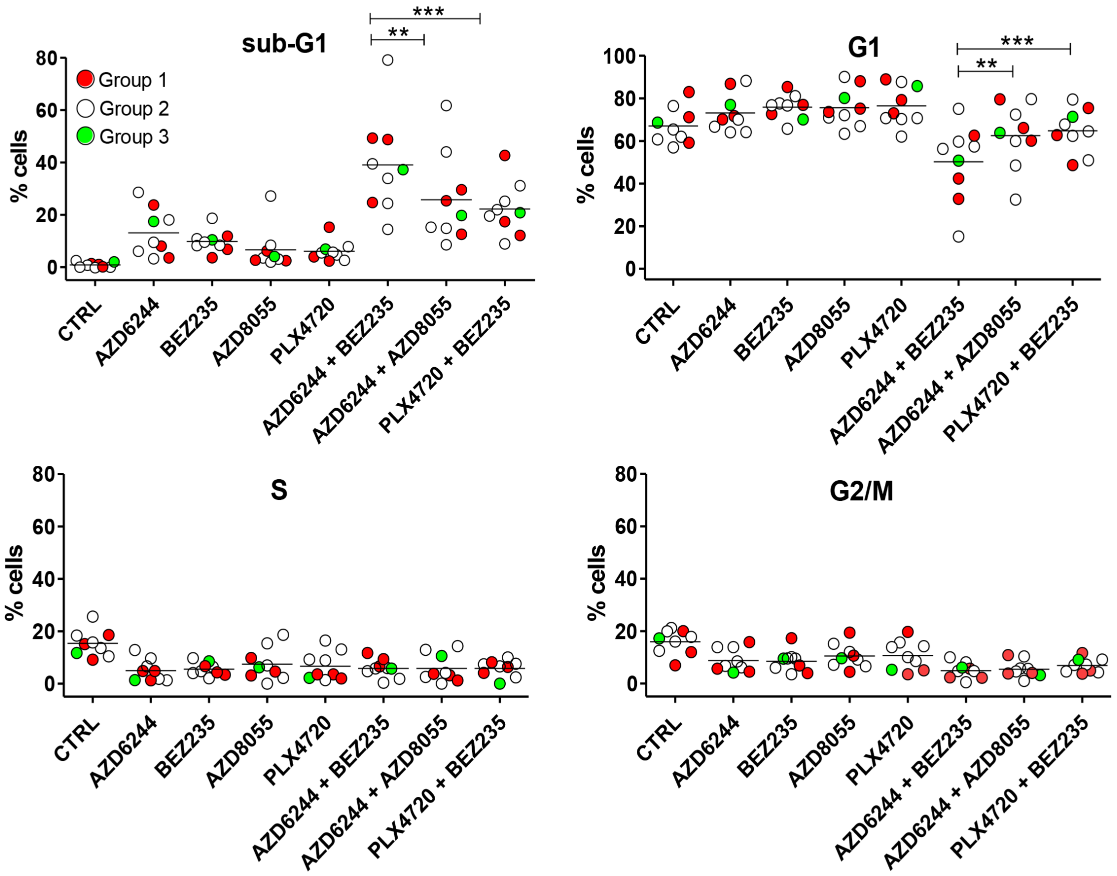

Supplementary Figure S9: The AZD6244-BEZ235 treatment promotes a significant increase in sub-G1 fraction compared to PLX4720-BEZ235 and to AZD6244-AZD8055 association. DNA content analysis, at 48 hours, of 9 melanoma cell lines cultured with BRAFV600E, MEK1/2, PI3K/mTOR inhibitors and their association. AZD6244, BEZ235 and AZD8055 were used at 0.2 µM; PLX4720 was used at 0.5 µM. Each point, in each panel, indicates % of cells of a single melanoma cell line belonging to the three susceptibility groups as defined in Figure 1. Group 1: Me13, Me6 and Me79; group 2: Me41, Me17, Me21, Me45 and Me63 ; Group 3: Me71. The four panels indicate the % of cells in each of the cell cycle phases (G1, S, G2M) and in the sub-G1 fraction. Statistical analysis by ANOVA followed by SNK test. ***$p < 0.001$; **$p < 0.01$.

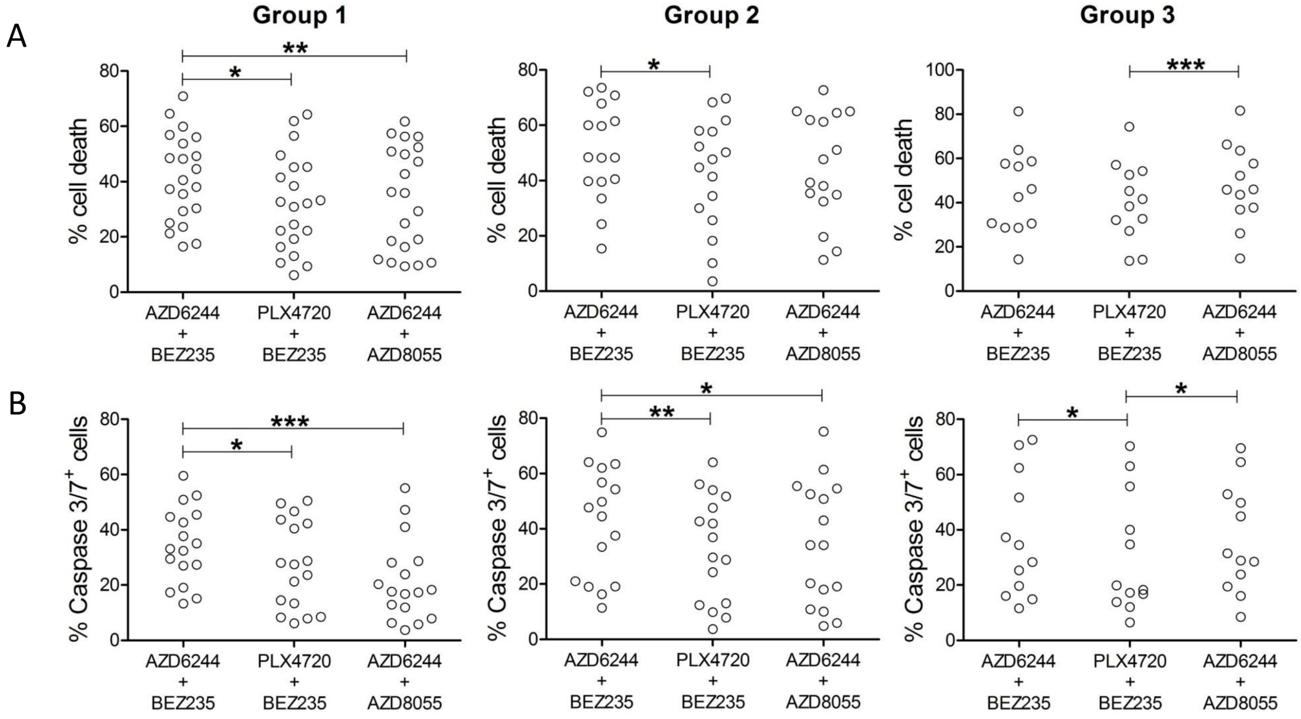

Supplementary Figure S10:Promotion of cell death and caspase 3/7 activation by dual blockade of MEK1/2 and PI3K/mTOR or mutant BRAF and PI3K/mTOR pathways. (**A**, **B**) Melanoma cell lines belonging to the three groups defined in Figure 1 (group 1, $n = 21$; group 2, $n = 16$; group 3, $n = 12$) were treated with the indicated combinations of inhibitors. AZD6244, BEZ235 and AZD8055 were used at 0.2 µM; PLX4720 was used at 0.5 µM. Cell death (A) was assessed at 72 h and caspase 3/7 activation (B) at 48 h by the MUSE cell analyzer. Statistical analysis by repeated measure ANOVA followed by SNK test. ***$p < 0.001$, **$p < 0.01$.

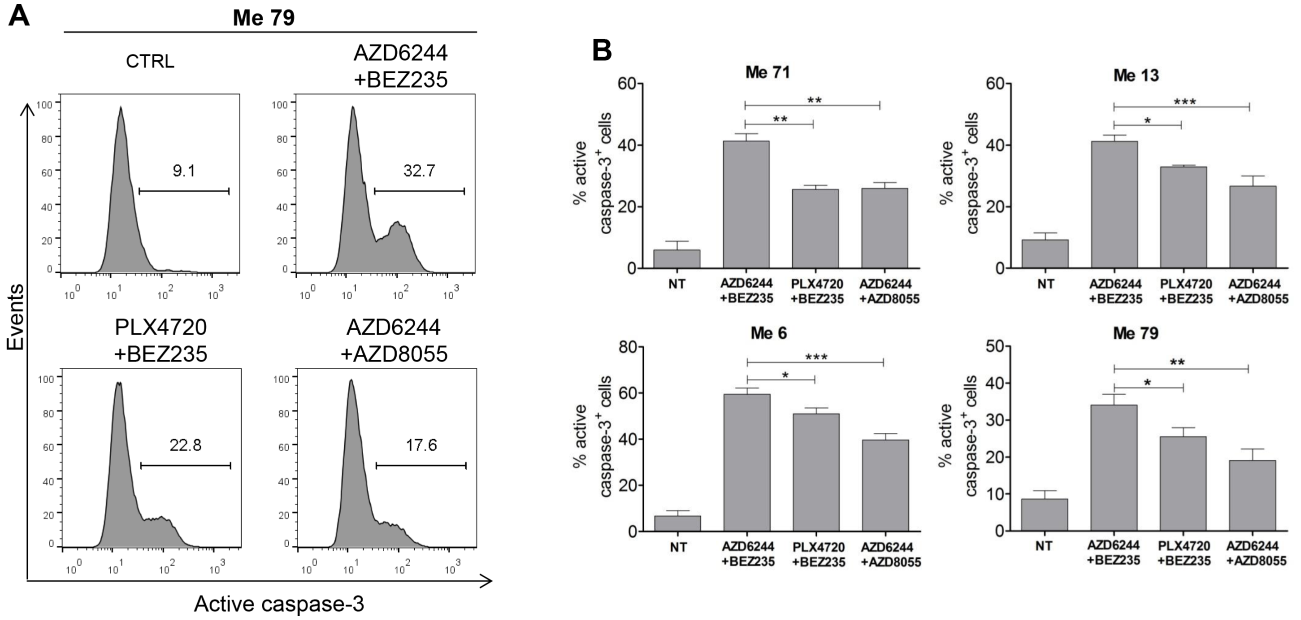

Supplementary Figure S11:Combinatorial treatments promote activation of caspase-3. (**A**, **B**) Melanoma cell lines belonging to group 1 (Me13, Me6 and Me79) or to group 3 (Me71) were treated with the indicated associations of inhibitors for 48 h. Inhibitors doses as in the legend to Figure 7. Cells were then stained with anti-active caspase-3 antibody. (A) Representative data of active caspase-3 staining in Me79 cells after treatment with the indicated combinations of inhibitors. Numbers in each panel: % positive cells, markers set based on staining with isotype control (B) Results from five independent experiments. Statistics by one-way ANOVA, followed by Newman-Keuls post-test. *$p < 0.05$, **$p < 0.01$, ***$p < 0.001$.

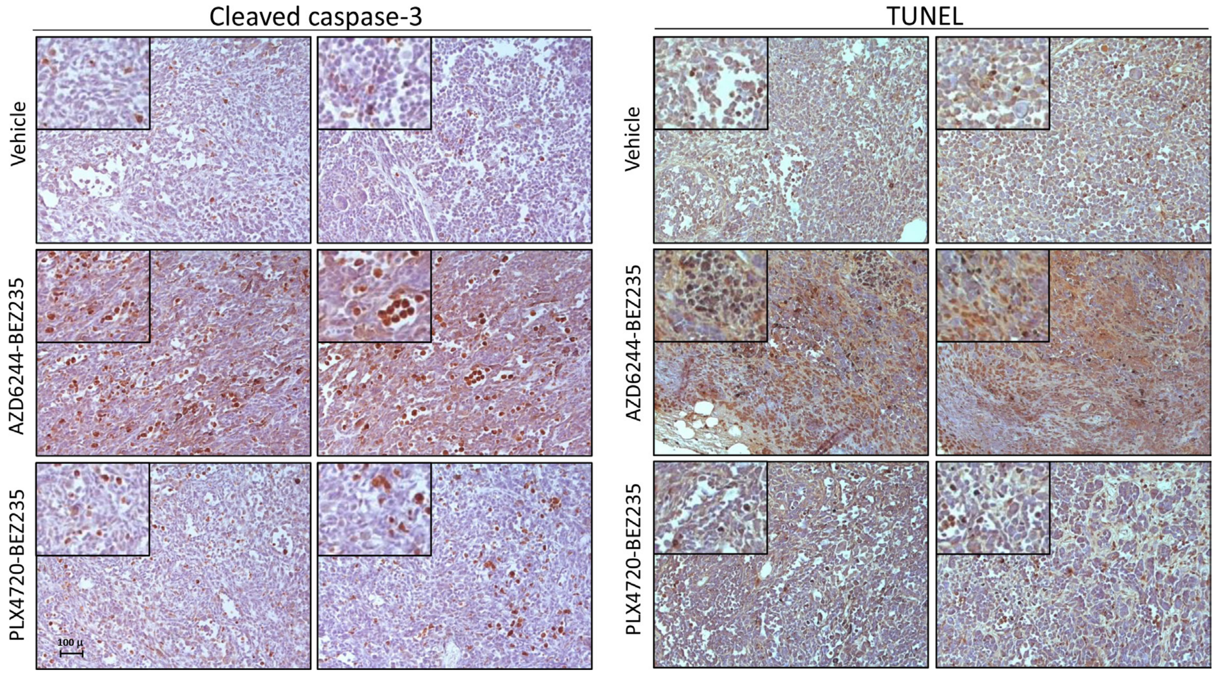

Supplementary Figure S12 :Activation of caspase-3 and promotion of melanoma apoptosis *in-vivo* by combinatorial treatments. Staining with anti- active, cleaved caspase-3 antibody and with TUNEL of tumor nodules (images of nodules from two animals are shown) removed after the last administration of inhibitors (day 31), from control mice (vehicle) and from mice treated with the association of AZD6244-BEZ235 or of PLX4720-BEZ235. Insets, higher magnification of a representative area of each panel. Original magnification, 20×.

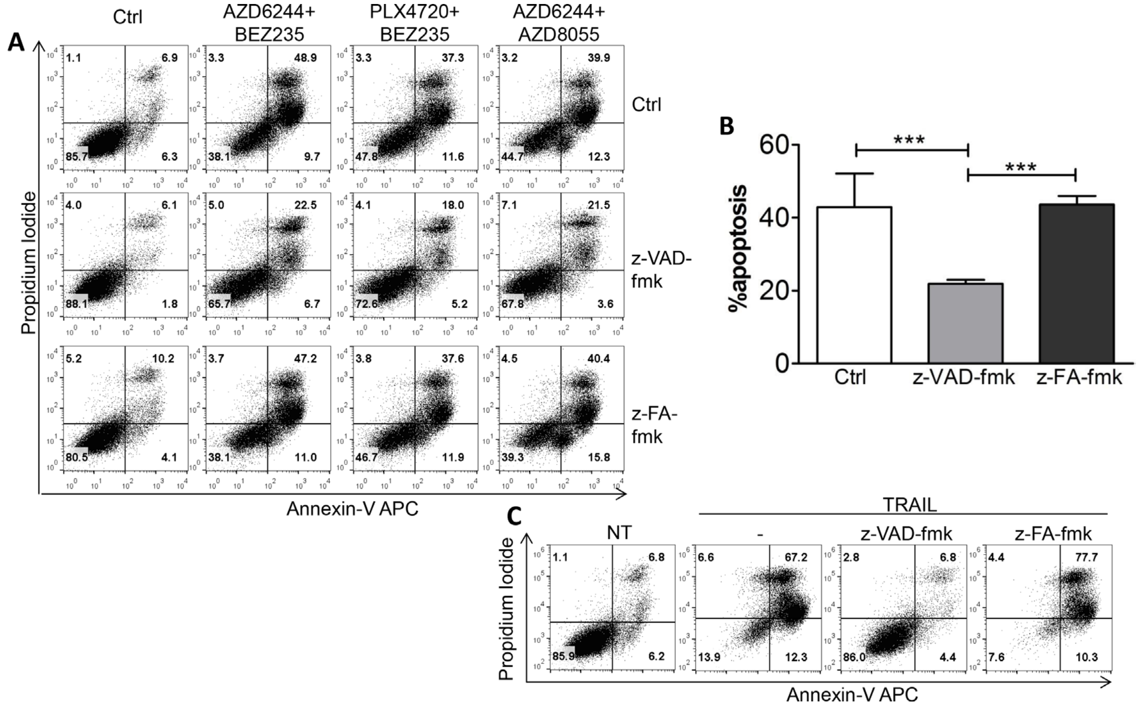

Supplementary Figure S13 :Enhanced melanoma apoptosis by combinatorial treatments is caspase-dependent. (**A)** Annexin-V/Propidium Iodide stainings in Me6 cells treated or not with the association of AZD6244 (0.1 μM) and BEZ235 (0.1 μM), PLX4720 (0.5 μM) and BEZ235 (0.1 μM), or AZD6244 (0.1 μM) and AZD8055 (0.05 μM) for 48 h. Cells were pre-incubated with 5 μM of the pan-caspase inhibitor z-VAD-fmk or of its negative control z-FA-fmk. Numbers in the dotplots: % cells in each quadrant. (**B**) Me6 were treated with AZD6244-BEZ235 as in A and pre-incubated with z-VAD-fmk or z-FA-fmk. Results of the Annexin-V/Propidium Iodide stainings (sum of ANN+/PI- and ANN+/PI+ cells) are the mean of three independent experiments. Statistical analysis by ANOVA and SNK test. ***$p < 0.001$ (**C**) Me6 cells, preincubated with z-VAD-fmk or z-FA-fmk, were treated with recombinant TRAIL (100 ng/ml) for 48 h, as a positive control of caspase-dependent apoptosis.

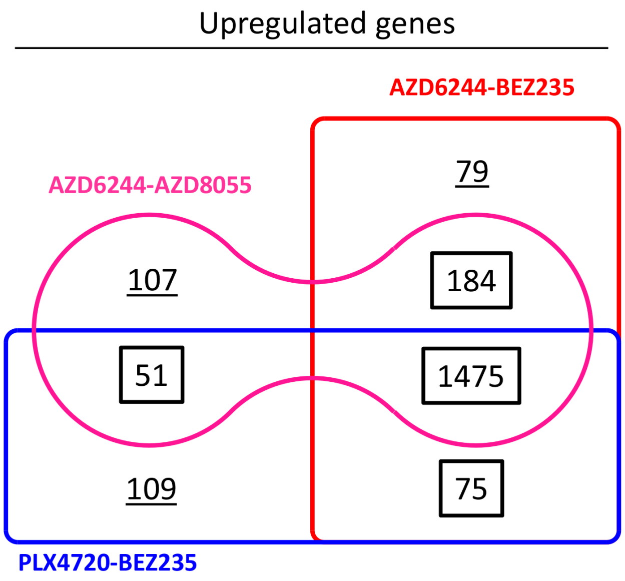

Supplementary Figure S14 :Edwards-VENN diagram analysis of genes modulated in Me13 melanoma cells upon dual blockade of MEK1/2 and PI3K/mTOR or mutant BRAF and PI3K/mTOR pathways. Upregulated genes (left diagram) and downregulated genes (right diagram) by treatment with AZD6244-BEZ235 (red rectangle), PLX4720-BEZ235 (blue rectangle) or AZD6244-AZD8055 (fuchsia doughnut shape) compared to control, untreated cells. Underlined values: combination-specific gene expression changes. Boxed values: shared gene expression changes among different combinatorial treatments. Significantly modulated genes (by BRB-array Tools, Vers.4.3.0) were identified by class comparison carried out by a random-variance F-test with a nominal significance level of 0.001, as previously described (18, 38). Permutation *P* values for significant genes were computed based on 10,000 random permutations. Pairwise analysis of significance of gene modulation between any two of the treatments was carried out at $P = 0.01$.

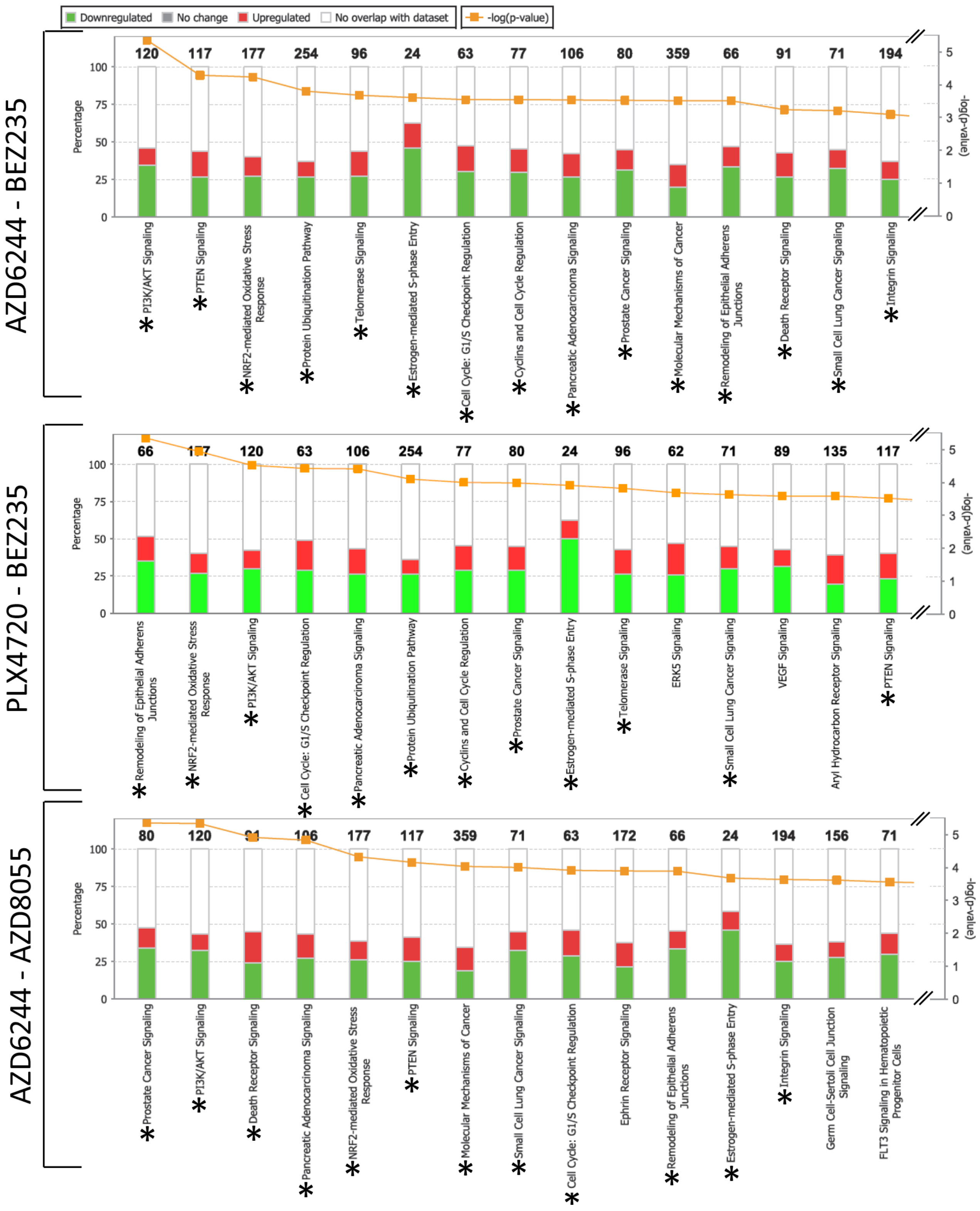

Supplementary Figure S15 :Top 15 canonical pathways involved in the modulation of gene expression by three different combinatorial treatments, as identified by IPA analysis on significantly modulated genes. Significance values [-log(*p*-value)] for the canonical pathways (values plotted on the right Y axis) calculated by Fisher's exact test right- tailed. Significance indicates probability of association of molecules with the canonical pathway by random chance alone. The percentage (plotted on the left axis as stacked bar charts) indicates the fraction of genes in the pathway that are downregulated (green), or upregulated (red), or that have no overlap with dataset (white). *Shared canonical pathways, among the top 15, found to be affected by at least two or all three combinatorial treatments.

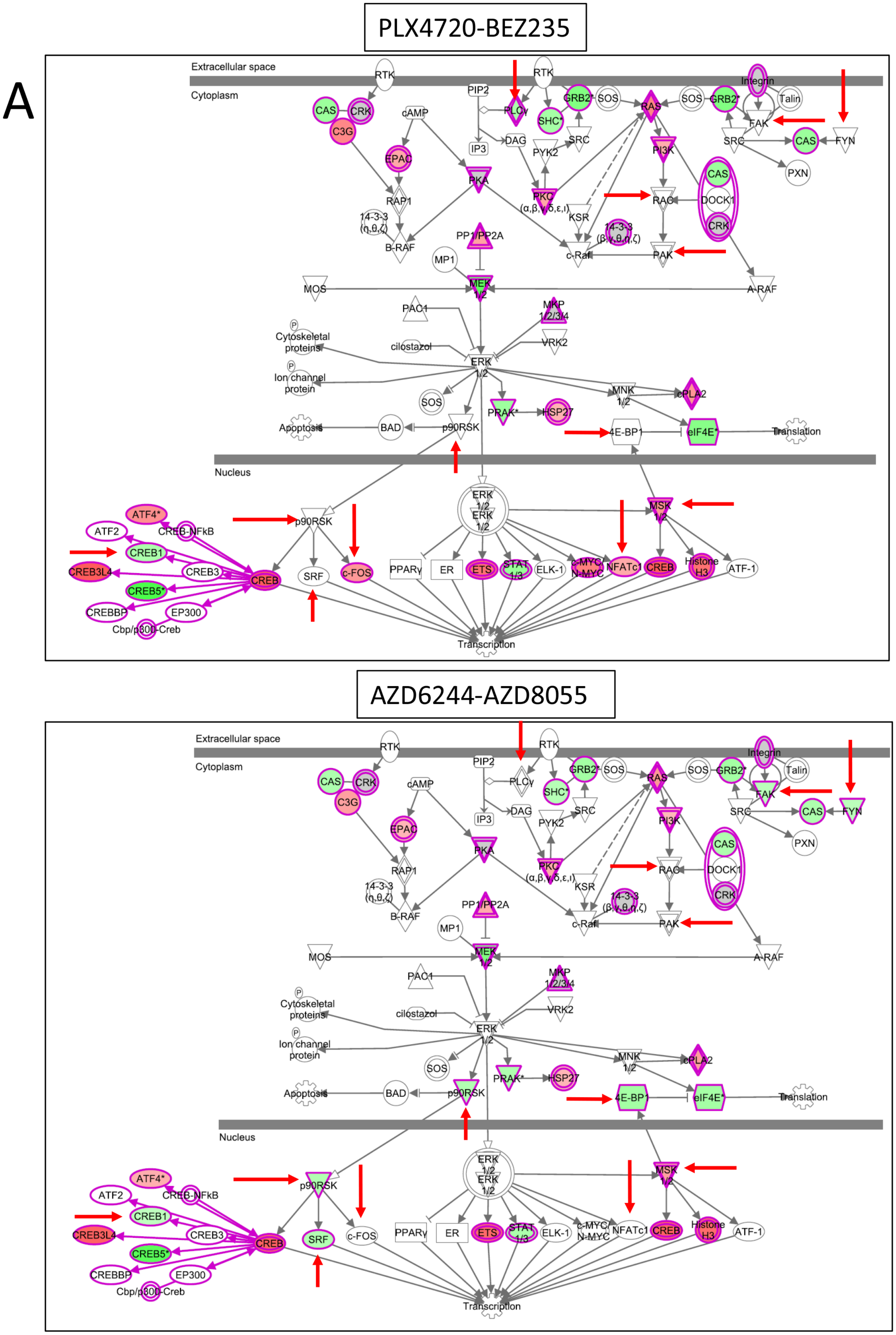
A
PLX4720-BEZ235
AZD6244-AZD8055
Extracellular space
Cytoplasm
Nucleus
RTK
PIP2
IP3
DAG
cAMP
PLCγ
SHC*
GRB2*
SOS
RAS
Integrin
Talin
FAK
SRC
CAS
FYN
PXN
CRK
C3G
EPAC
PKA
RAP1
14-3-3 (η,θ,ζ)
B-RAF
PYK2
PKC (α,β,γ,δ,ε,ι)
PI3K
RAC
DOCK1
KSR
14-3-3 (β,γ,θ,η,ζ)
c-Raf
PAK
A-RAF
PP1/PP2A
MP1
MOS
MEK 1/2
PAC1
MKP 1/2/3/4
VRK2
Cytoskeletal proteins
cilostazol
ERK 1/2
Ion channel protein
SOS
MNK 1/2
cPLA2
PRAK*
HSP27
4E-BP1
eIF4E*
Translation
Apoptosis
BAD
p90RSK
MSK 1/2
ATF4*
ATF2
CREB-NFkB
CREB1
CREB3
CREB3L4
CREB5*
CREB
CREBBP
EP300
Cbp/p300-Creb
SRF
c-FOS
PPARγ
ER
ETS
STAT 1/3
ELK-1
c-MYC N-MYC
NFATc1
Histone H3
ATF-1
Transcription

B

PLX4720-BEZ235

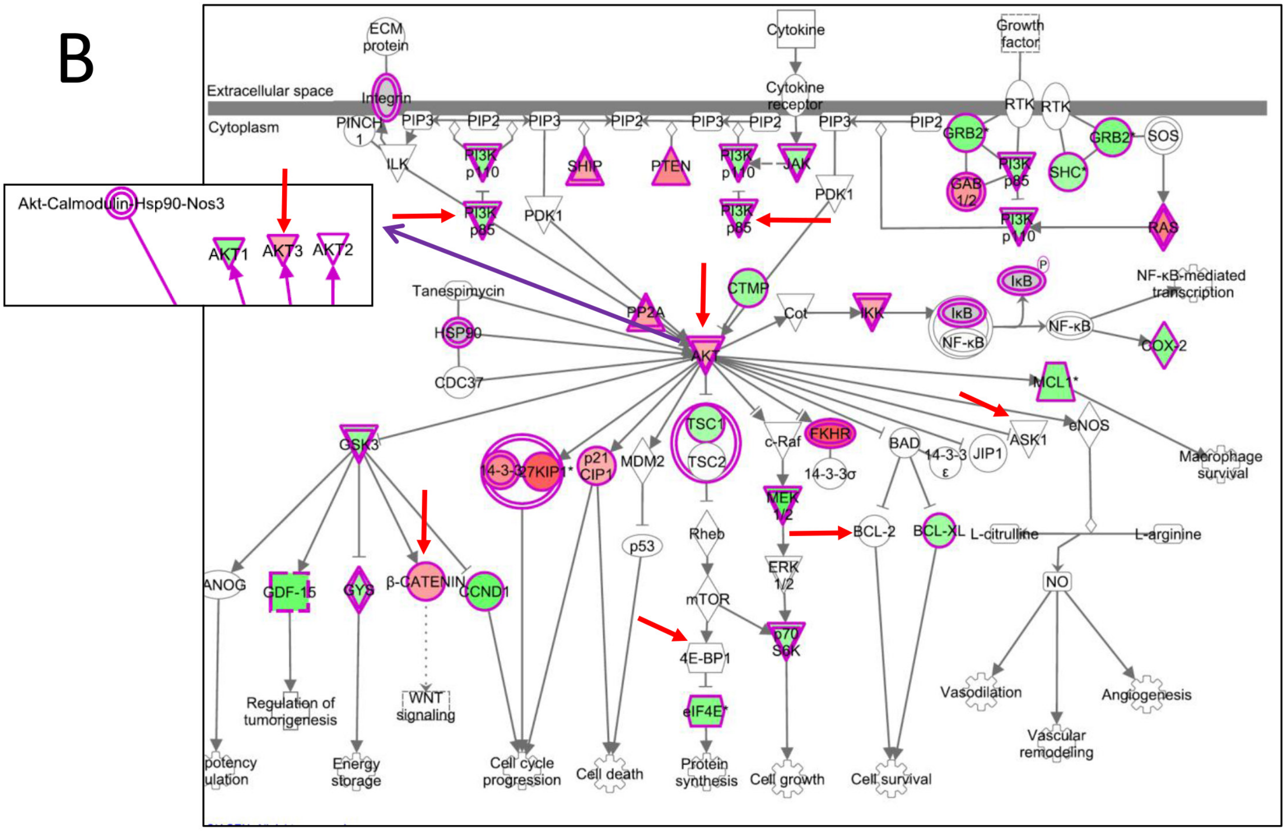

AZD6244-AZD8055

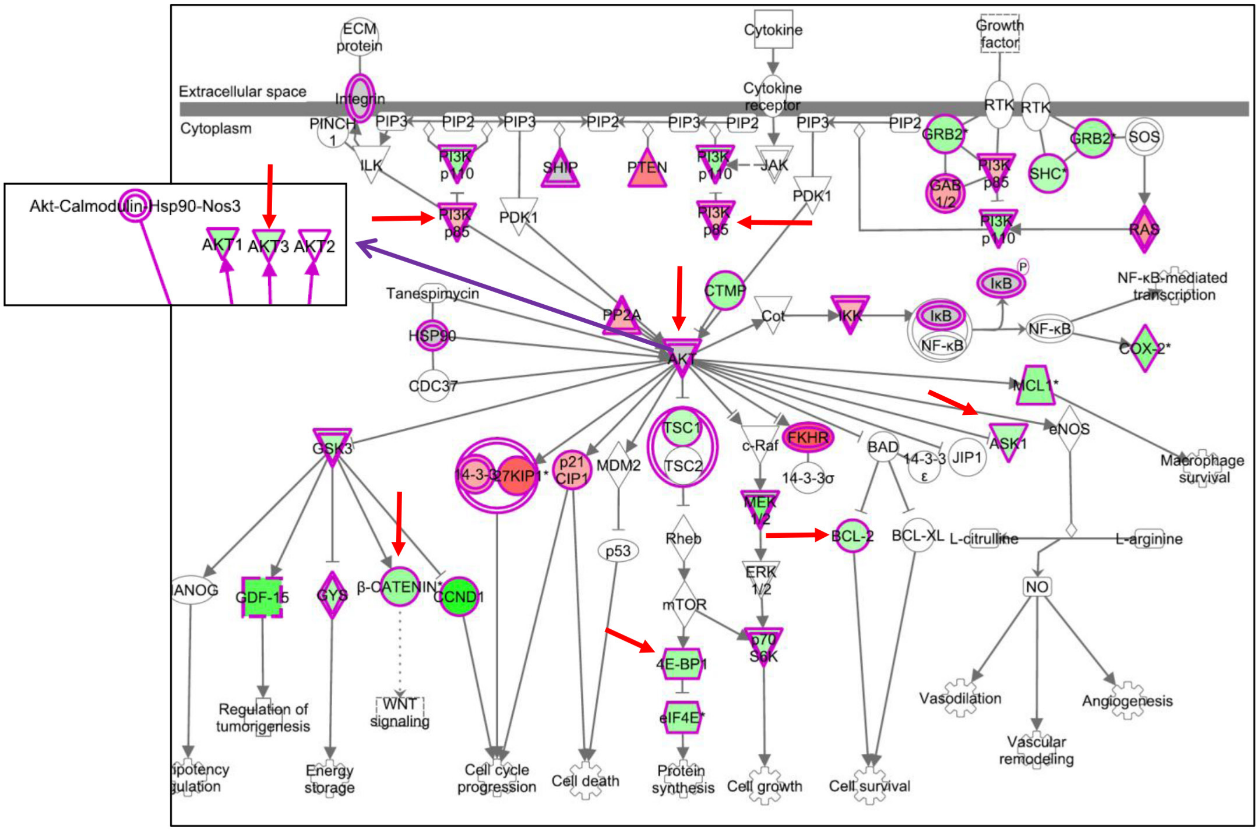

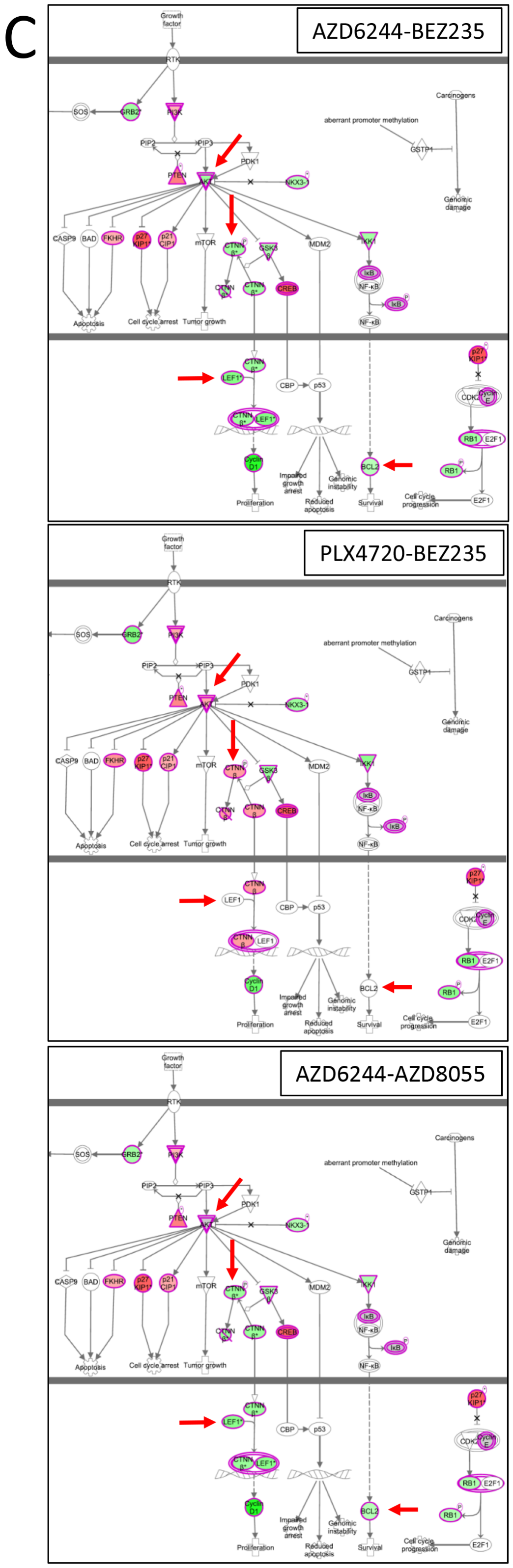

Supplementary Figure S16 :Modulation of genes in the ERK/MAPK, PI3K/AKT and "prostate cancer signaling" canonical pathways by combinatorial treatments. (**A, B**) genes modulated by PLX4720- BEZ235 and AZD6244-AZD8055 associations in the ERK/MAPK canonical pathway (**A**) and in the PI3K/AKT canonical pathway (B). (**C**) Genes modulated by AZD6244-BEZ235, PLX4720-BEZ235 and AZD6244-AZD8055 associations in "Prostate cancer signaling canonical pathway." Upregulated genes are shown in red, and downregulated genes in green. Red arrows: genes differently affected by AZD6244-BEZ235 compared to PLX4720-BEZ235 and/or to AZD6244-AZD8055 combinatorial treatments (compare panels A and B with Figure 10A and 10B, respectively).

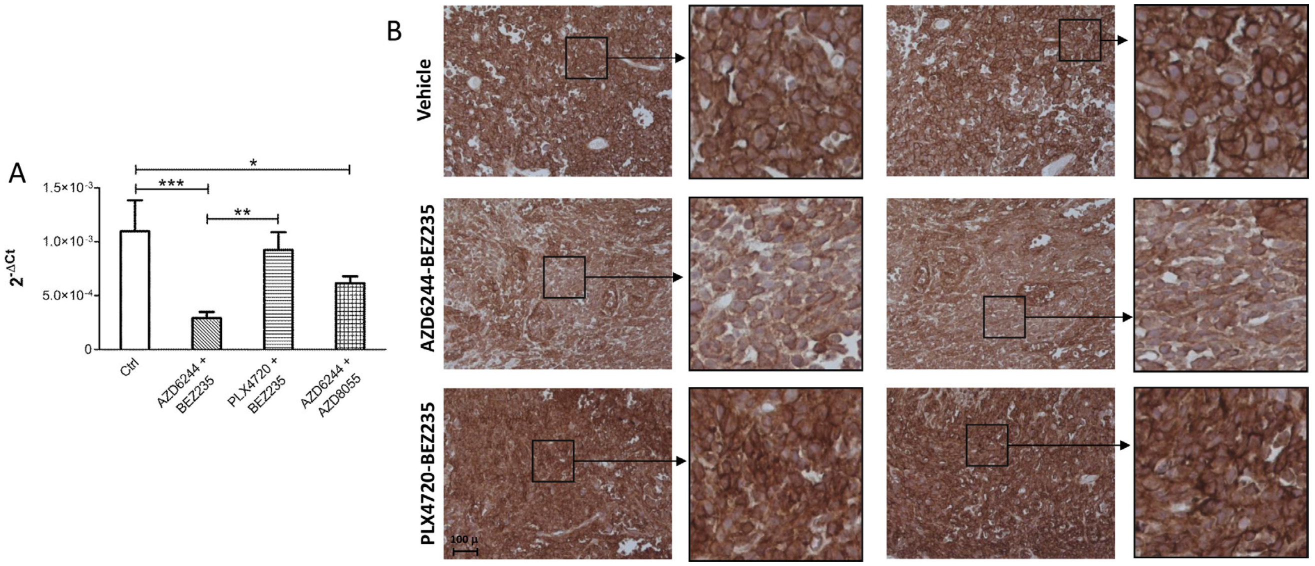

Supplementary Figure S17 :Differential modulation of c-FOS and P catenin by combinatorial treatments. (**A**) qPCR analysis for c-FOS mRNA levels in Me13 cells treated with AZD6244-BEZ235, PLX4720-BEZ235 or AZD6244-AZD8055 combinations. Results expressed by the arbitrary unit of expression $2^{-\Delta Ct}$ as described in Materials and methods. Statistical analysis by ANOVA and SNK test. ***$p < 0.001$, **$p < 0.01$, *$p < 0.05$. (**B**) Immunohistochemistry analysis for P catenin in tumor nodules (images of nodules from two animals are shown) removed after the last administration of inhibitors from control mice treated with vehicle and from mice treated with the association of AZD6244-BEZ235 or of PLX4720- BEZ235, as described in the legend to Supplementary Figure 5. Insets, higher magnification of a representative area of each panel. Original magnification, 20×.
